# A bispecific Clec9A-PD-L1 targeted type I interferon profoundly reshapes the tumor microenvironment towards an antitumor state

**DOI:** 10.1186/s12943-023-01908-6

**Published:** 2023-11-29

**Authors:** Sandra Van Lint, Alexander Van Parys, Bram Van Den Eeckhout, Niels Vandamme, Stephane Plaisance, Annick Verhee, Dominiek Catteeuw, Elke Rogge, Jennifer De Geest, Nele Vanderroost, Jana Roels, Yvan Saeys, Gilles Uzé, Niko Kley, Anje Cauwels, Jan Tavernier

**Affiliations:** 1 Center for Medical Biotechnology, VIB & Department of Biomolecular Medicine, Ghent University, Ghent, Belgium; 2 Cancer Research Institute Ghent (CRIG), Ghent, Belgium; 3 Present Affiliation: Orionis Biosciences, Ghent, Belgium; 4 VIB Single Cell Core, VIB, Ghent-Leuven, Belgium; 5 Department of Biomedical Molecular Biology, Ghent University, Ghent, Belgium; 6 VIB Nucleomics Core, Leuven, Belgium; 7 Data Mining and Modelling for Biomedicine, VIB & Center for inflammation research, Ghent University, Ghent, Belgium; 8 Department of Applied Mathematics, Computer Science and Statistics, Faculty of Science, Ghent University, Ghent, Belgium; 9 IRMB, University Montpellier, INSERM, CNRS, Montpellier, France; 10 Orionis Biosciences, Ghent, Belgium; 11 Orionis Biosciences, Boston, USA

**Keywords:** Type I Interferon, AcTakine, Tumor microenvironment, Cancer immunotherapy, Immune checkpoint

## Abstract

Despite major improvements in immunotherapeutic strategies, the immunosuppressive tumor microenvironment remains a major obstacle for the induction of efficient antitumor responses. In this study, we show that local delivery of a bispecific Clec9A-PD-L1 targeted type I interferon (AcTaferon, AFN) overcomes this hurdle by reshaping the tumor immune landscape.

Treatment with the bispecific AFN resulted in the presence of pro-immunogenic tumor-associated macrophages and neutrophils, increased motility and maturation profile of cDC1 and presence of inflammatory cDC2. Moreover, we report empowered diversity in the CD8^+^ T cell repertoire and induction of a shift from naive, dysfunctional CD8^+^ T cells towards effector, plastic cytotoxic T lymphocytes together with increased presence of NK and NKT cells as well as decreased regulatory T cell levels. These dynamic changes were associated with potent antitumor activity. Tumor clearance and immunological memory, therapeutic immunity on large established tumors and blunted tumor growth at distant sites were obtained upon co-administration of a non-curative dose of chemotherapy.

Overall, this study illuminates further application of type I interferon as a safe and efficient way to reshape the suppressive tumor microenvironment and induce potent antitumor immunity; features which are of major importance in overcoming the development of metastases and tumor cell resistance to immune attack. The strategy described here has potential for application across to a broad range of cancer types.

**Supplementary Information:**

The online version contains supplementary material available at 10.1186/s12943-023-01908-6.

## One sentence summary

Treatment with type I interferon simultaneously targeting Clec9A- and PD-L1-expressing cells profoundly reshapes the immune landscape leading to immunological memory, therapeutic immunity and clearance of large established tumors when combined with low-dose chemotherapy.

## Introduction

The immunosuppressive tumor microenvironment exerts a plethora of inhibitory mechanisms, creating a major obstacle to cancer immunotherapy [1]. One such mechanism is defective antigen-presentation resulting in inefficient tumor-antigen (cross-)presentation towards T cells [2]. Tumor-associated antigen-presenting cells (APC) might also contribute to a suppressive or tolerogenic state resulting in T cell silencing rather than T cell stimulation [2]. Moreover, several immune cell types including pro-tumorigenic tumor-associated macrophages (TAM) and regulatory T cells (Treg) are key regulators of immunosuppression, tumor-promoting angiogenesis and the formation of metastases [3]. On the level of T cells, the induced and tumor-infiltrating tumor-specific cytotoxic T lymphocytes (CTL) often fail to exercise antitumor effector functions [4]. Furthermore, antitumor immune responses are often curtailed due to overexpression of inhibitory immune checkpoint molecules such as Programmed Death-Ligand 1 (PD-L1) [5]. Current cancer immunotherapeutic strategies are therefore strongly focusing on promoting antitumor immunity while simultaneously overcoming tumor-induced immune suppression.

Due to its pleiotropic role, type I interferon (IFN) [6] might be key to promote antitumor immunity and to overcome tumor-induced immune suppression [7]. Despite its considerable potential, clinical application of type I IFN has been impeded due to significant systemic toxicities, which are a consequence of ubiquitous expression of the type I IFN receptor complex [8].

As an answer to the need for safe and adequate cancer treatments, we are developing ‘AcTakines’, Activity-on-Target Cytokines, consisting of mutated cytokines coupled to a specific targeting domain. As such, AcTakines only exert potent activity on targeted cells, while avoiding pleiotropic systemic cytokine activity and associated toxicities [9]. We already provided evidence for the broad applicability of AcTakines in cancer immunotherapy [10–13], auto-immunity [14, 15] and as an adjuvant in prophylactic vaccination strategies such as influenza [16].

In this study, we elaborate on a type I IFN AcTakine (AcTaferon, AFN) simultaneously targeting Clec9A and PD-L1. The protein-engineered AcTakine presented here includes a mutated version of the IFN-α2 sequence. The choice for Clec9A as a targeting moiety that delivers type I IFN activity towards cross-presenting type I cDC (cDC1) was made in view of our earlier work, which described potent antitumor immunity obtained upon treatment with this AcTakine [10]. Clec9A encodes for DNGR-1 [17], which represents a C-type lectin receptor with an expression profile that’s restricted to cDC1s in human [17], making it an ideal therapeutic target for selective delivery of payload and subsequent triggering of cDC1 [18].

PD-L1 is a co-inhibitory ligand for the Programmed Death receptor (PD-1) and is constitutively expressed or induced on many immune cells as well as on various cancer cell types. Under normal physiological conditions, the PD-1/PD-L1 interaction is essential for the development of immune tolerance and prevents excessive activation of the immune system or immune exhaustion. However, cancer cells in particular use PD-L1 as an immune evasion mechanism [19]. Over the years, immune checkpoint blockade agents that interfere with the PD-1/PD-L1 pathway have gained significant importance as anticancer immunotherapeutics.

Here, we demonstrate that treatment with a bispecific Clec9A-PDL1 targeted AcTaferon induces tumor control accompanied with changes at the level of various cell types including myeloid cells, DCs, antigen-specific CTLs, increased presence of NK and NKT cells as well as decrease of tumor-resident Tregs. Moreover, in combination with a non-curative dose of chemotherapy, tumor clearance, immunological memory, therapeutic immunity against large established tumors and blunted tumor growth at distant sites were observed. Strikingly, the bispecific AcTaferon was able to reshape the suppressive tumor microenvironment towards an antitumor state and induced TCR epitope spreading.

## Results

### Superior tumor control by bispecific AcTaferon with high safe profile

**Fig. 1:**
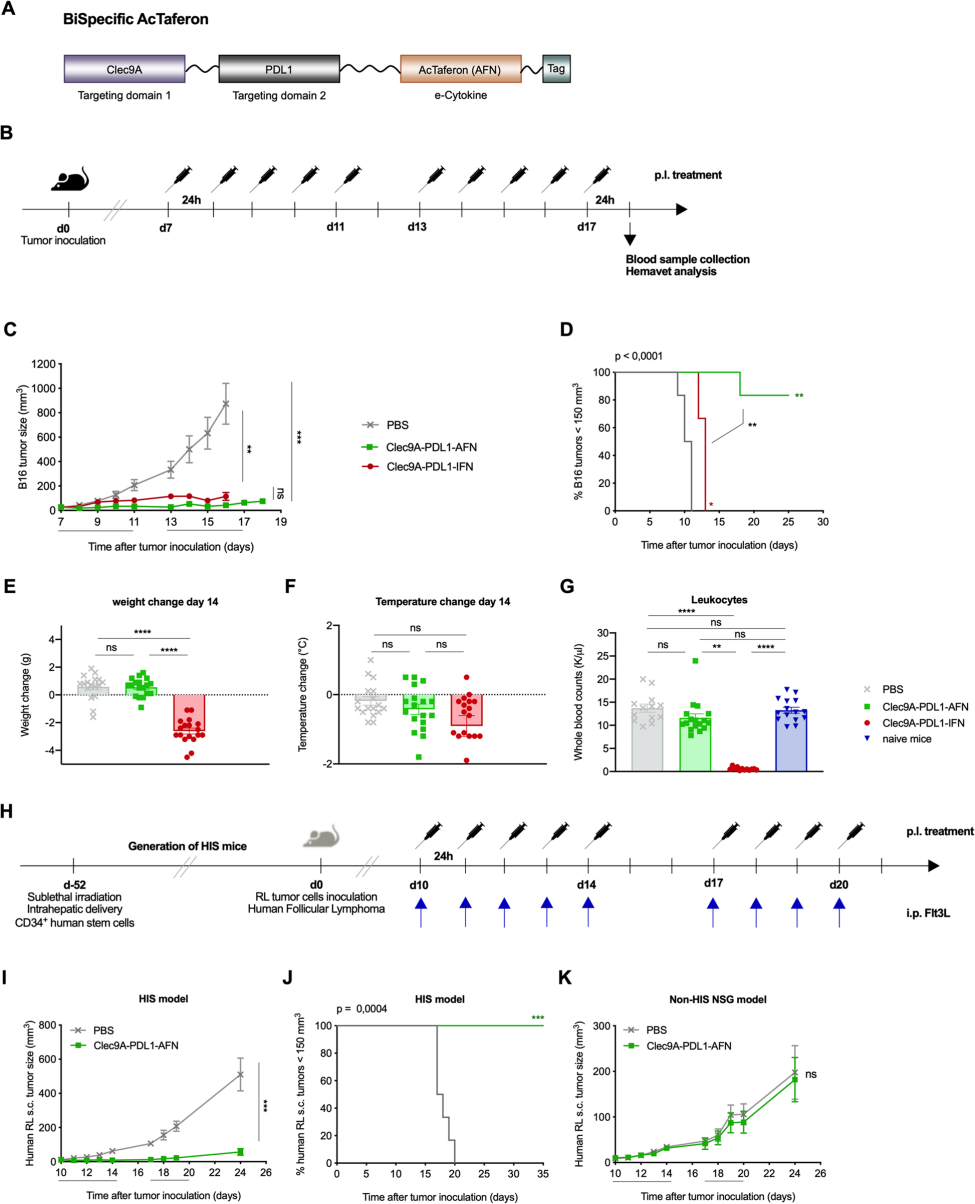
Tumor control by Bispecific AcTaferon shows ample potential and is completely safe. **A** Design and layout of the Bispecific AcTaferon (AFN) construct. Targeting domains are N-terminally connected to the mutated cytokine. A HIS-tag was added for easy purification. **B** Schematic representation of the experimental setup. Mice were s.c. inoculated with 6 × 10^5^ B16 tumor cells. When palpable tumor was detected (day 7 after tumor inoculation) mice were treated 10 times with a perilesional administration with PBS (grey), 30 μg of the Bisp-AFN Clec9A-PDL1-AFN (green) or 30 μg of the Clec9A-PDL1-IFN wild type (red) according to the indicated arrows above the timeline. **C**, **D** Figures show tumor growth (**C**) as well as time to reach a B16 tumor volume of 150 mm^3^ (**D**). One representative experiment out of three is shown (6/group/experiment). **E**–**F** Weight change (**E**) and body temperature (**F**) are depicted at day 14 after B16 tumor inoculation. Values are relative to day 7, representing the start of the treatment schedule. The graphs show individual values ± SEM of 3 independent experiments (6/group/experiment). **G** One day after the last treatment in the B16 model, blood was collected, and hematological analysis (Hemavet) was performed. Leukocyte analysis is shown as a summary of individual values ± SEM of three independent experiments (6/group/experiment). **H** Schematic representation of the experimental layout using a Humanized Immune System (HIS) mouse model. Mice were s.c. inoculated with RL tumor cells. When palpable tumor was detected mice were treated with a perilesional administration with PBS (grey) or 30 μg of the fully humanised Bisp-AFN Clec9A-PDL1-AFN (green) according to the indicated arrows above the timeline. Intraperitoneal administration of Flt3L is indicated by the blue arrows underneath the timeline. **I**-**J** Figures show tumor growth (**I**) as well as time to reach an RL tumor volume of 150 mm^3^ (**J**) in an HIS setting. **K** The graph shows RL tumor growth in absence of a human immune system (Non-HIS NSG mice). One representative experiment out of two is shown (5–6/group/experiment). Tumor growth (**C**) was analyzed using Two-way ANOVA with Tukey’s multiple comparisons test. For the HIS/non-HIS model (**I**, **K**), tumor growth was analyzed at day 24 using unpaired two-tailed student t-test. Black lines underneath the X-axis depict the treatment time. Time to reach a specific tumor size (**D**, **J**) is represented in a Kaplan Meier plot compared by log-rank (Mantel-Cox) test. Bar plots (**E**, **F**, **G**) were analyzed using One-way ANOVA Kruskal–Wallis test with Dunn’s multiple comparisons test. * < 0.05; ** < 0.01; *** < 0.001; **** < 0.0001

Here, we aim to investigate whether simultaneous targeting of type I IFN activity towards Clec9A and PD-L1 can induce antitumor efficacy (Fig. 1A-B). In first instance, we evaluated the antitumor potential in different mouse tumor models including B16 (Fig. 1C-D) as well as 4T1 mammary carcinoma, both subcutaneously (s.c.) (Suppl. Fig. 1A-B) and orthotopically implanted (Suppl. Fig. 1C-D). Administration of the bispecific Clec9A-PDL1-AFN didn’t affect body weight, body temperature nor analysed blood parameters in the 4T1 model (data not shown). Treatment with the Bisp-AFN resulted in B16 tumor stasis comparable to wild type (wt) IFN (Fig. 1C-D). However, wt IFN caused severe body weight loss (Fig. 1E), a decrease in body temperature although not statistically significant (Fig. 1F), and affected all blood parameters monitored (Fig. 1G, Suppl. Fig. 1E) causing anemia, lymphopenia, leukopenia and platelet destruction, which resulted in high mortality. In sharp contrast to this, treatment with Bisp-AFN was very well tolerated.

To bring the strategy closer to clinical application, we additionally tested our findings in a humanized setting. To that end, both Humanized Immune System (HIS) mice and irradiated non-HIS NSG mice were inoculated with RL cells, a human non-Hodgkin B cell lymphoma (Fig. 1H). The antitumor potential of the humanized Bisp-AFN was favorable and resulted in delayed tumor growth after therapy termination (Fig. 1I-J). Absence of a response in non-HIS NSG mice indicated the need for an active immune system, rather than the direct anti-proliferative effects of type I IFN on the tumor (Fig. 1K).

### AcTaferon therapy combined with doxorubicin leads to tumor cure, therapeutic immunity and abscopal systemic effects

**Fig. 2:**
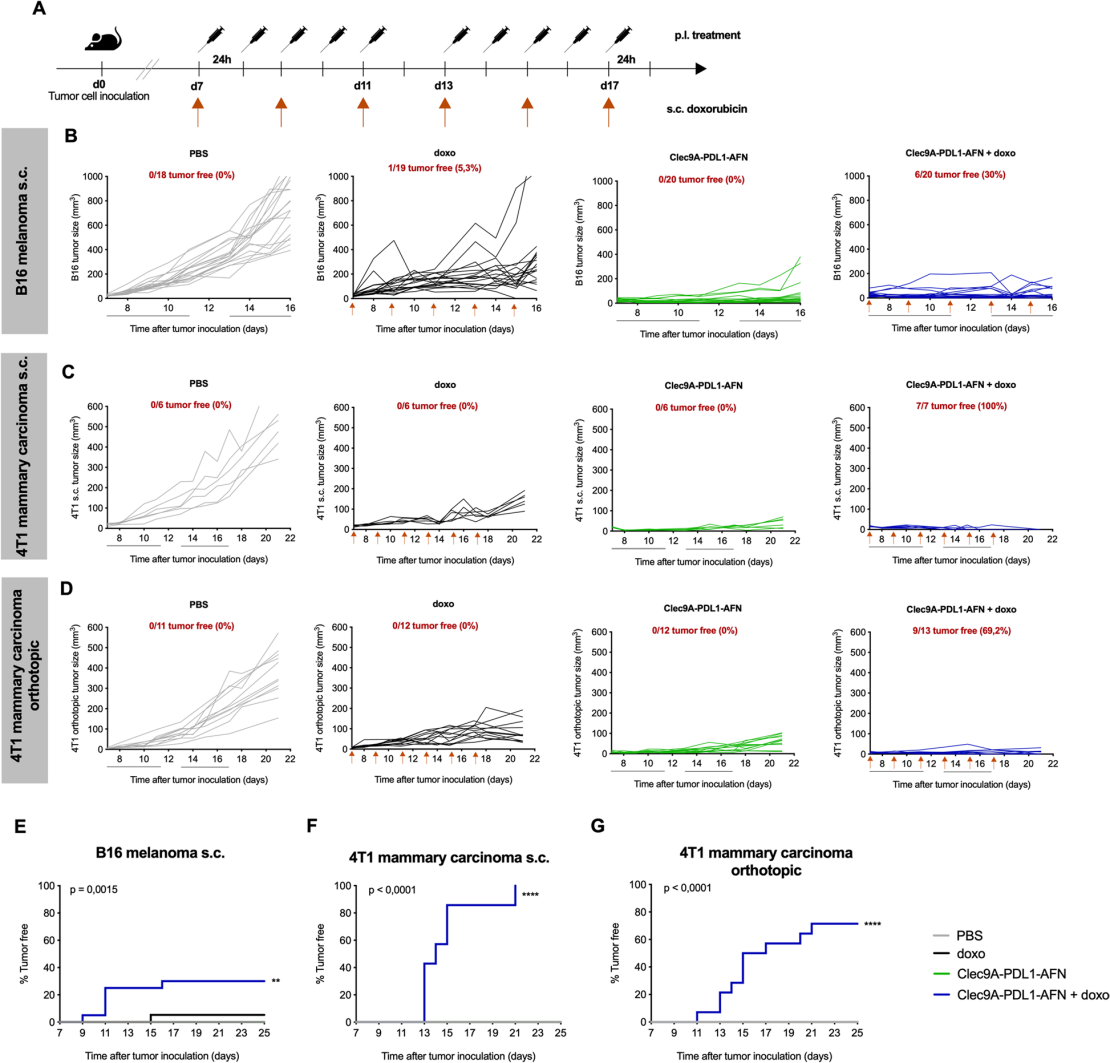
Combination therapy with doxorubicin leads to complete tumor cure. **A** Schematic representation of the experimental setup. **B**, **E** B16 melanoma cells were s.c. inoculated. Results show a summary of three independent experiments (6–8/group/experiment). **C**, **F** 4T1 mammary carcinoma cells were s.c. inoculated. Shown is one experiment (*n* = 6). **D**, **G** Orthotopic 4T1 mammary carcinoma model. Results show a summary of two independent experiments (6/group/experiment). Tumor growth progression is depicted for the individual mice in each group (**B**-**D**). Kaplan Meier graphs (**E**–**G**) show tumor free mice. When palpable tumors were detected, mice were treated with PBS (grey), doxo (black), 30 μg of Bisp-AFN Clec9A-PDL1-AFN (green) or a combination of Clec9A-PDL1-AFN with doxo (blue). Black lines underneath the X-axis depict the treatment time, while orange arrows indicate s.c. administration of doxo. Kaplan Meier plots depicting % tumor free mice (**E**–**G**) were analyzed using log-rank (Mantel-Cox) test with * *p* < 0.05; ** *p* < 0.01; *** *p* < 0.001; **** *p* < 0.0001

As evidenced in the cancer immunity cycle, establishment of durable antitumor immunity results from a cyclic process, which can be fueled at different levels using strategic interventions [20]. Combined treatment with immunogenic chemotherapeutics might be an interesting strategy (Fig. 2A), as we have shown before [10]. Combination of Bisp-AFN with a non-curative dose of doxorubicin (doxo) resulted in promising effects. In the B16 melanoma tumor model, which is considered to be non- or low-immunogenic, we observed a cure rate of 30% (6/20) (Fig. 2B, E). The outcome was even more striking in the 4T1 mammary carcinoma model. In the s.c. model, we observed a 100% cure (Fig. 2C, F). Also in an orthotopic setting, which mimics the biological and metastatic tumor cell properties observed in clinical cancer patients [21], promising cure rates were observed (70%) (Fig. 2D, G).

**Fig. 3:**
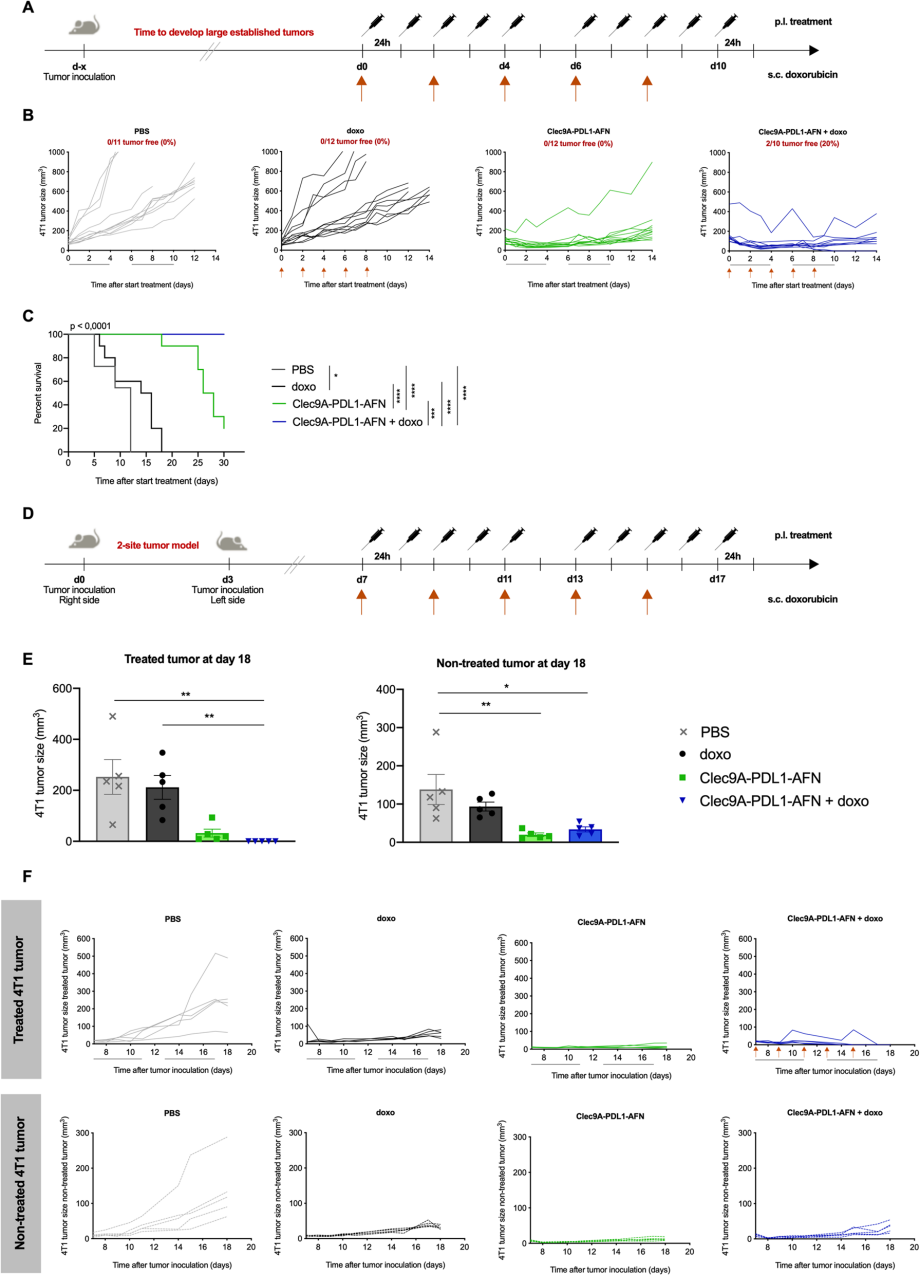
Combination therapy with doxorubicin leads to therapeutic immunity and systemic effects. **A** Schematic representation of the experimental setup. Treatment was started when large established tumors were reached (120–150 mm^3^). **B**, **C** Figures show 4T1 tumor growth of individual mice in each group (**B**) as well as survival (**C**) (one experiment was performed with *n* = 12/group). **D** Schematic representation of the experimental setup for a two-site tumor model. Primary tumors were inoculated at the right flank followed by a second tumor inoculation at the contralateral flank. Primary tumors were p.l. treated whereupon systemic effects of local treatment were analyzed on disseminated non-treated tumors (left flank). **E** The graphs show s.c. 4T1 tumor volume of individual values ± SEM at day 18 (*n* = 5). **F** Tumor growth curves of both treated (upper panel) and non-treated (lower panel) tumors of individual mice using the s.c. 4T1 mammary carcinoma model. Mice were treated with PBS (grey), doxo (black), 30 μg of Bisp-AFN Clec9A-PDL1-AFN (green) or a combination of Clec9A-PDL1-AFN with doxo (blue). Black lines underneath the X-axis depict the treatment time, while orange arrows indicate s.c. administration of doxo. Kaplan Meier plots depicting % survival (**C**) were analyzed using log-rank (Mantel-Cox) test. Bar plots (**E**) were analysed using One-way ANOVA Kruskal–Wallis test with Dunn’s multiple comparisons test (treated tumors) or using One-way ANOVA followed by bonferroni’s multiple comparison test (non-treated tumors) dependent on Shapiro–Wilk test for normal distribution of the data. * < 0.05; ** < 0.01; *** < 0.001; **** < 0.0001

In clinical settings, there is often an urgent need for adequate therapies against fast growing and fully developed tumors. Hence, we analyzed the antitumor potential of AFN and doxo therapy in large established tumors (Fig. 3A). Bisp-AFN with doxo could suppress and diminish tumor growth even after the treatment was stopped, resulting in prolonged survival (Fig. 3B-C). In addition, 20% were cured upon treatment with Bisp-AFN plus doxo.

Encouraged by these results, we set up a 2-site tumor model to analyze abscopal effects and systemic therapeutic immunity (Fig. 3D). Strikingly, treatment with doxo plus Bisp-AFN resulted in complete cure of the treated tumor and prolonged growth inhibition of the non-treated 4T1 tumor (Fig. 3E-F).

These results demonstrate that antitumor immune effects elicited by local immunomodulation upon treatment with Bisp-AFN in combination with non-curative doses of doxorubicin can induce systemic effects and affect tumor growth at distant sites.

### Induction of immunological memory by combined treatment of Bispecific AcTaferon with doxorubicin

**Fig. 4:**
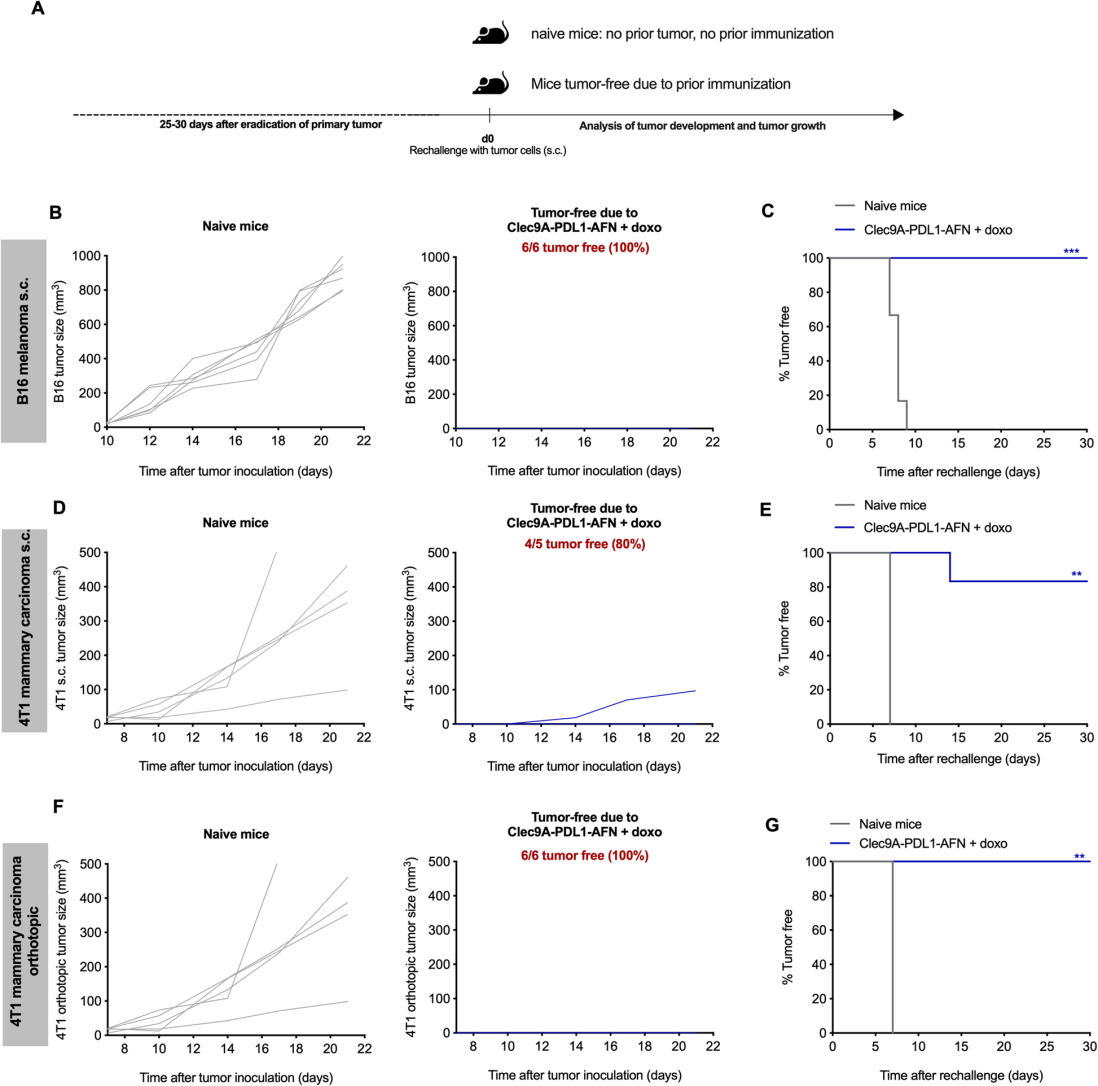
Induction of immunological memory upon combined treatment with doxorubicin. **A** Schematic representation of the experimental layout. Tumor-free mice over the different experiments were re-challenged s.c. in the contralateral flank on day 25–30 after eradication of the primary tumor. For each tumor model, mice without prior tumor inoculation nor treatment, referred to as ‘naive mice’, were included and injected with the indicated tumor cells as a control for disease progression. **B**-**E** Mice cured from an s.c. primary tumor were s.c. re-challenged with 6 × 10^5^ B16 cells (**B**-**C**) or 10^5^ 4T1 cells (**D**-**E**). **F**-**G** Mice in which the orthotopically implanted primary 4T1 tumor was completely cured were re-challenged s.c. with 10^5^ 4T1 cells. Figures show tumor growth measured of individual mice in each group (**B**, **D**, **F**) as well as % tumor free mice (**C**, **E**, **G**). Kaplan Meier graphs depicting % tumor free mice (**C**, **E**, **G**) were analyzed using log-rank (Mantel-Cox) test. * < 0.05; ** < 0.01; *** < 0.001; **** < 0.0001

Since combined therapy with doxo resulted in complete tumor eradication (Fig. 2A-G), we could evaluate the potential immunological memory of the treatments in the cured mice (Fig. 4A). Despite that only few tumors were cured in the B16 model (Fig. 2B, E), 100% (6/6) protective immunity was observed upon treatment with doxo plus the Bisp-AFN (Fig. 4B-C). In the s.c. 4T1 model, 80% (4/5) immunological memory was achieved in mice that had been cured following doxo plus Bisp-AFN treatment (Fig. 4D-E). The one individual that was not protected showed delayed tumor development and growth (Fig. 4D). Finally, in the 4T1 orthotopic model, we observed 100% (6/6) immunological memory that was achieved in mice that had been cured upon doxo plus Bisp-AFN treatment (Fig. 4F-G).

These data indicate the strong curative potential of Bisp-AFN and doxorubicin treatment, independent of the histological origin of the tumor.

### Role of IFN signaling and PD-L1 expression

**Fig. 5:**
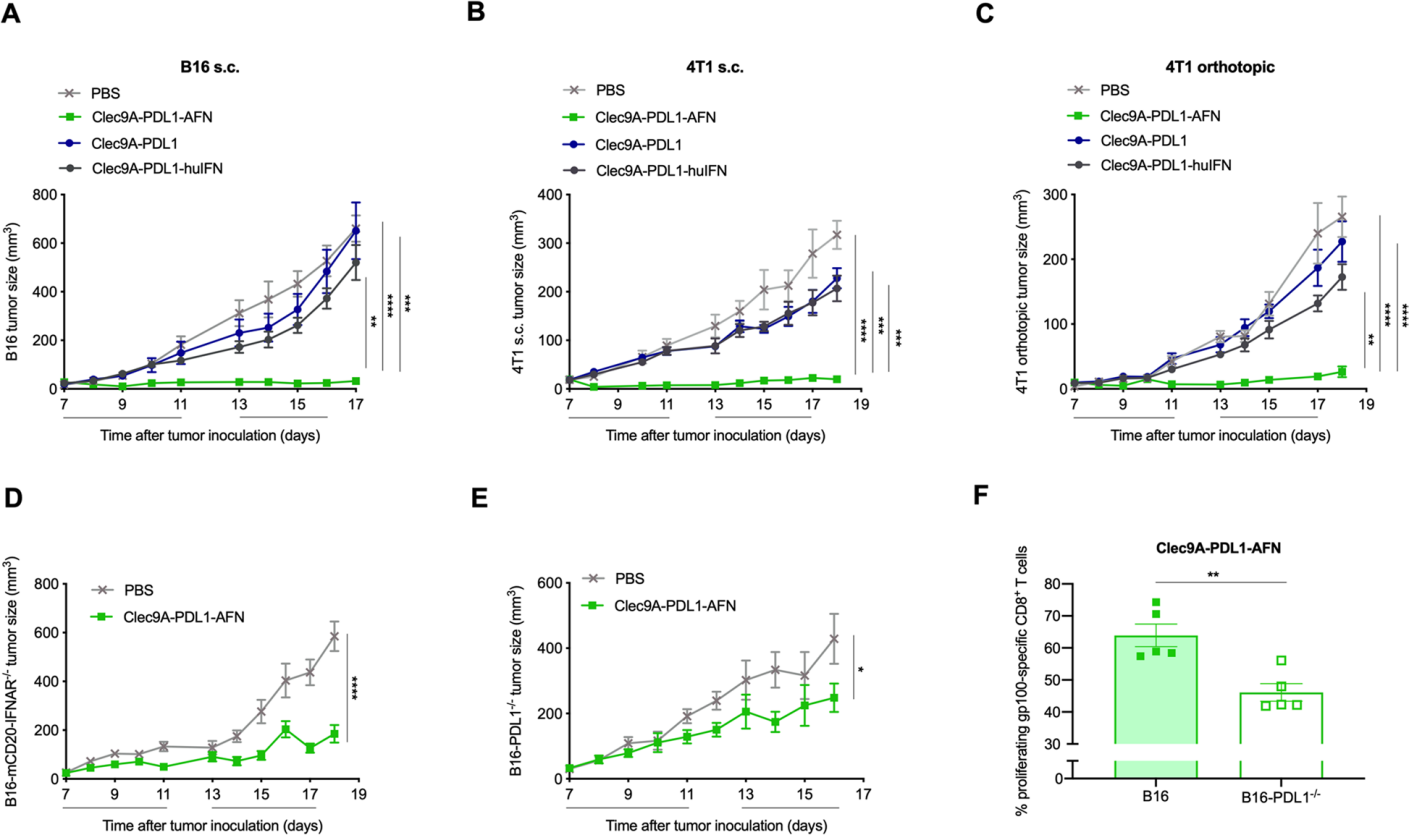
Role of IFN signaling and PD-L1 expression. **A**-**C** Tumor cells were s.c. or orthotopically inoculated. Tumors were p.l. treated with PBS (grey), 30 μg of the Bisp-AFN (green), 30 μg of the bispecific construct carrying an in mouse non-functional human IFN (blue) or 30 μg of the Clec9A-PDL1 construct, lacking an IFN moiety (dark grey). Figures show tumor growth in the s.c. B16 model (**A**) (one representative experiment out of three, 6mice/group/experiment), s.c. 4T1 tumor model (**B**) (*n* = 6) and the orthotopic 4T1 tumor model (**C**) (*n* = 6). **D** Tumor growth of B16-mCD20-IFNAR^−/−^ tumors p.l. treated with PBS (grey) or Bisp-AFN (green). Shown is a summary of two independent experiments (6/group/experiment). **E** Tumor growth of B16-PD-L1^−/−^ tumors p.l. treated with PBS (grey) or Bisp-AFN (green). Shown is a summary of two independent experiments (6/group/experiment). **F** Flow cytometry analysis of gp100-specific CD8^+^ T cell proliferation in draining LN of B16 or B16-PD-L1^−/−^ tumor-bearing mice p.l. injected with 30 μg Clec9A-PDL1-AFN at days 7 and 9 after tumor inoculation. One day prior to immunization, gp100-specific CD8^+^ T cells (pMel) were adoptively transferred in B16 tumor-bearing mice. Data show percentages of gp100-specific CD8.^+^ T cells that have undergone at least one division. Tumor growth (**A**-**C**) was analyzed using ANOVA with Tukey’s multiple comparisons test. Black lines underneath the X-axis depict the treatment time. Bar charts show individual values with mean ± SEM. Two-tailed unpaired t test was performed. * < 0.05; ** < 0.01; *** < 0.001; **** < 0.0001

In contrast to the bispecific Clec9A-PDL1-AFN, administration of Clec9A-PDL1-huIFN, which cannot signal in mouse cells, hardly showed any antitumor efficacy against B16 melanoma (Fig. 5A), in the 4T1 mammary carcinoma s.c. (Fig. 5B) or orthotopic (Fig. 5C) model. These data were additionally confirmed by administration of Clec9A-PDL1, a bispecific control construct without an IFN moiety (Fig. 5A-C). Altogether, these findings indicate that the pure tethering effect might be insufficient and that IFN signaling is key for the robust antitumor efficacy. Next, we analyzed antitumor responses in B16-mCD20-IFNAR^−/−^ tumors, lacking a functional IFN-α and -β Receptor subunit 1 (IFNAR1). Potent antitumor responses were observed with the Bisp-AFN (Fig. 5D), indicating that IFN signaling in tumor cells is not needed for the antitumor effect upon administration of the BiSp-AFN. These data suggest that IFN signaling in immune cells rather than direct intrinsic and anti-proliferative effects on tumor cells is key for the observed effects. In contrast to IFNAR expression by tumor cells, PD-L1 expression by the tumor cells was needed for the antitumor efficacy of Bisp-AFN (Fig. 5E). These results were confirmed in a tumor antigen-specific proliferation assay using gp100 in a B16 melanoma model. To that end, CFSE labeled CD8^+^ T cells carrying a TCR specifically recognizing the melanoma differentiation antigen gp100 were adoptively transferred into B16 melanoma bearing mice. After treatment, dilution of CFSE was analyzed in draining LN. Proliferation was significantly impaired in the B16-PD-L1^−/−^ tumor model following Bisp-AFN therapy (Fig. 5F).

Besides the importance of co-localized interferon signaling in immune cell populations, we additionally suggest a role for PD-L1 expression by the tumor, which contributes to the strong and potent antitumor potential of Bisp-AFN treatment.

### Single cell RNA sequencing indicates key shifts in both lymphoid and myeloid cells in the tumor microenvironment

**Fig. 6:**
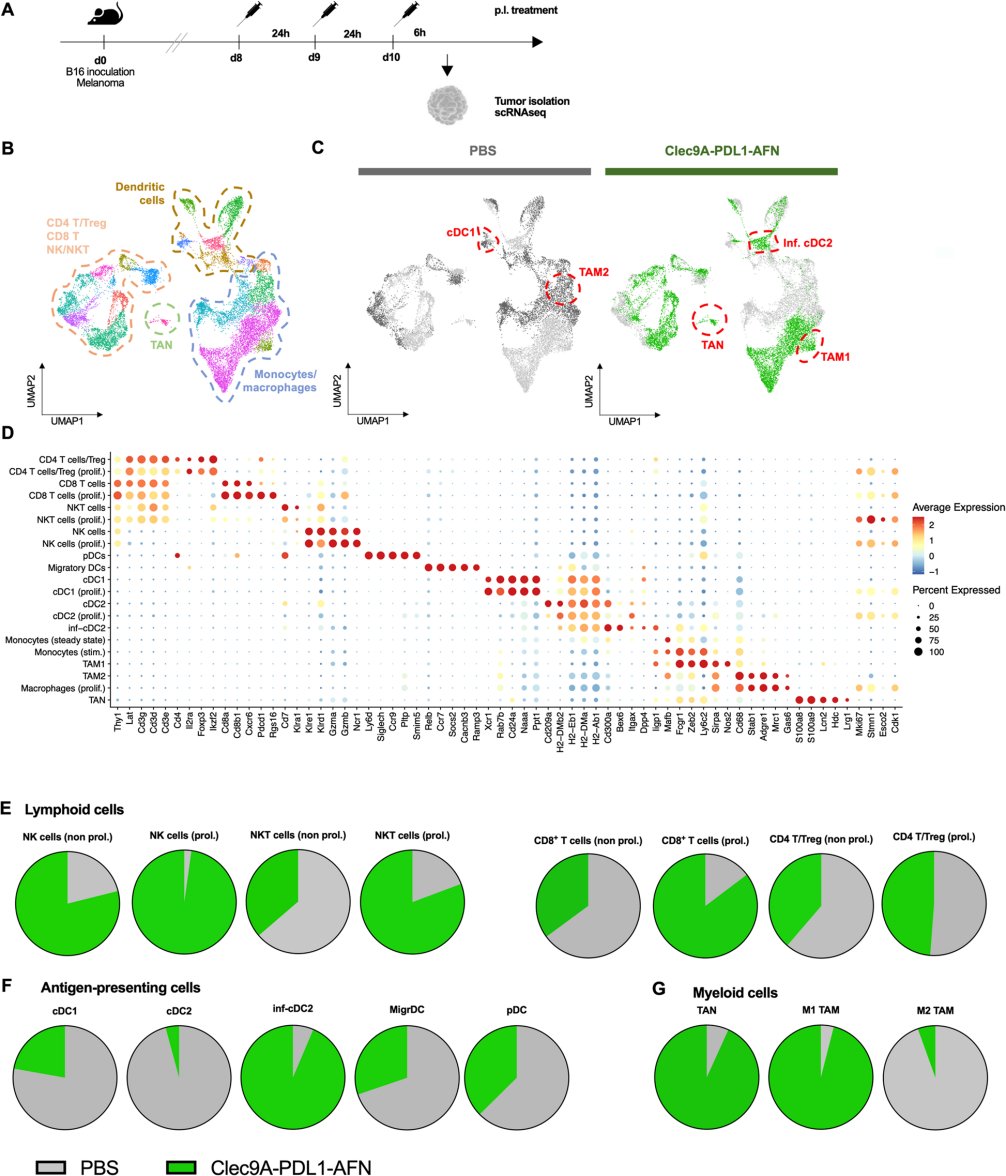
scRNAseq reveals key shifts in both lymphoid and myeloid cells in the tumor microenvironment. **A** Schematic representation of the scRNA sequencing experiment. Data obtained upon administration of PBS are represented in grey while data obtained after Clec9A-PDL1-AFN administration are visualized in green. **B**, **C** UMAP visualization showing the overall CD45^+^ living cell population in B16 tumors (**B**) as well as for the individual treatment groups (**C**). **D** Annotation plot showing differentially expressed (DE) genes in the X-axis to determine the different clusters (Y-axis). The size of the dot indicates the number of cells that express the gene of interest. The color intensity reflects the expression level (red = high, blue = low). **E**–**G** Parts of whole graphs showing relative distribution of immune cell types over the different treatments

To obtain a comprehensive understanding of the immune cell heterogeneity in the tumor microenvironment as well as effects induced by AcTaferon treatment, we performed single cell RNA sequencing (scRNAseq) on B16 tumor samples (Fig. 6A). Uniform Manifold Approximation and Projection (UMAP) for dimension reduction visualization was performed on the CD45^+^ living immune cell population. Cells from the various treatment conditions were pooled in a single dataset (Fig. 6B) whereupon the origin of each cell was visualized in a color-coded UMAP and linked to the different treatment conditions (Fig. 6C). Clusters were identified based on detection of differentially expressed (DE) genes (Fig. 6D, Suppl. Fig. 2, Suppl. Table 1). Based on these data, we could provide detailed information on the immune cell composition of B16 tumors (Suppl. Fig. 3). In addition, these scRNAseq data revealed key shifts in several immune cell compartments.

Therapy with the Bisp-AFN resulted in strong proliferation in the lymphoid compartment (NK cells, NKT cells and T cells) compared to PBS (Fig. 6E). Interestingly, tumors treated with Bisp-AFN showed less regulatory T cells compared to PBS (Fig. 6E).

On the level of APC, different DC subtypes were determined based on expression of DE genes (Suppl. Fig. 4A, Suppl. Table 1). The majority of cDC were detected in the PBS condition rather than in the Bisp-AFN condition (Fig. 6F). As cDC maturation and motility are tightly regulated, we presume that at the time-point of scRNAseq analysis (Fig. 6A) cDC in the Bisp-AFN condition already migrated from the tumor towards the draining LN. Indeed, flow cytometry analysis under the same conditions showed decreased amounts of cDC in B16 tumors after AFN treatment compared to PBS, while an increase was observed in draining LNs (Suppl. Fig. 4B). Besides cDC1, cDC2, migratory DC (migrDC) and pDC, scRNAseq data showed the presence of an additional DC population, which mainly arose in the Bisp-AFN treated condition (Fig. 6F). Based on the expression of *Fcgre1, Cd300a, Mafb, Bex6, Dnajc6* and *Ly6c2,* this population could represent monocyte-derived cells. However, additional genes including *Dpp4, Irf8, Sirpa, March1* as well as *Rsad2, Iigp1, Stat1, Ifit1, Ifit3* and *Ifi205* could be detected (Suppl. Table 1). Identification of these DE genes suggests that these cells could be an inflammatory cDC2 (inf-cDC2) population, as described by Bosteels et al. [22]. Inf-cDC2 arise from cDC2 upon inflammation and share phenotype, gene expression and function with both cDC1s and monocyte-derived cells [22]. Indeed, based on UMAP representation, this inf-cDC2 population is more closely related to cDC1 and cDC2 compared to the cluster of monocytes/macrophages (Fig. 6B-C). While only a negligible amount (6,4%) could be detected in the PBS condition, this inf-cDC2 population was most abundant in the Bisp-AFN condition (93,6%) (Fig. 6F).

Finally, clear differences between the different treatment conditions were detected on other cells of the myeloid compartment (Fig. 6G).

### From pro-tumorigenic towards pro-immunogenic TAMs and TANs

It is well known that the tumor microenvironment is a complex heterogeneity of tumor cells, stroma and a variety of infiltrated immune cell types [23]. Among these, tumor-associated neutrophils (TAN) and macrophages (TAM) comprise two noteworthy subtypes. To underscore this, our scRNAseq data in B16 tumors revealed key changes in these subtypes.

**Fig. 7:**
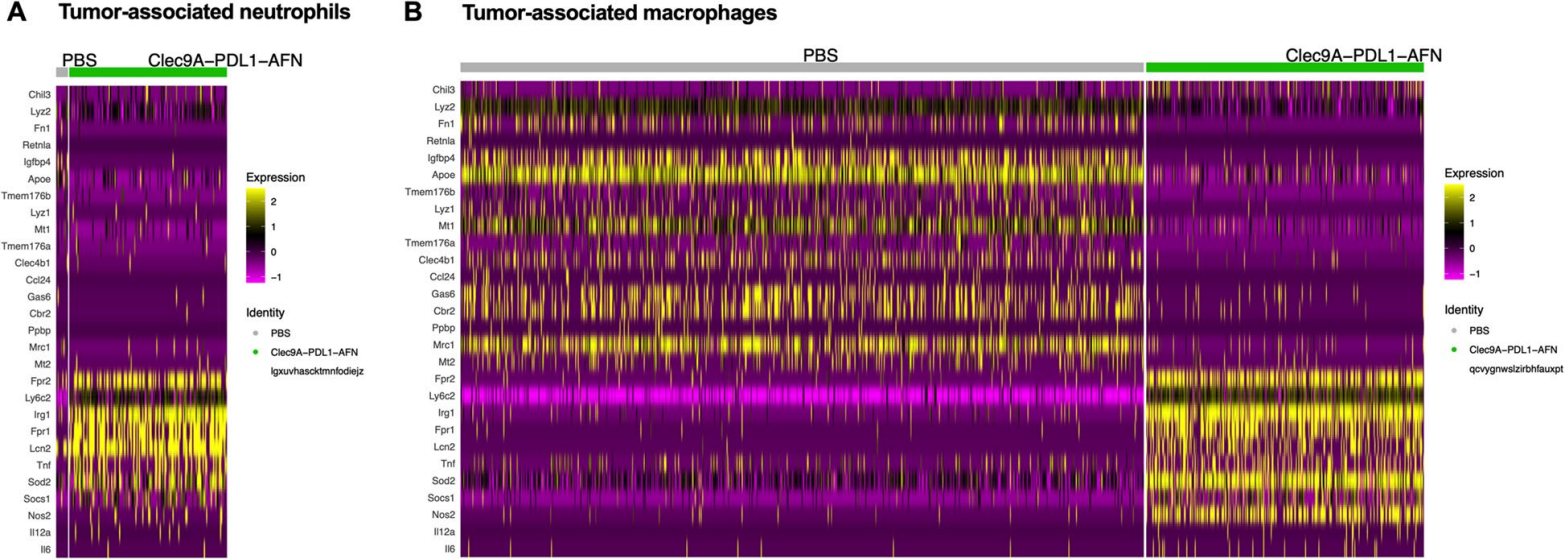
scRNAseq data on myeloid cells in the B16 tumor microenvironment. **A**, **B** Heatmap plots displaying differentially expressed genes related to an immunogenic or tumorigenic phenotype within TAN (**A**) and TAM (**B**)

Although neutrophils infiltrate in numerous cancer types, only low numbers were detected here. The identified neutrophils did not only almost selectively belong to the Bisp-AFN treated condition (Fig. 6G), they also showed a gene expression profile that is correlated with a pro-immunogenic TAN phenotype (Fig. 7A, Suppl. Fig. 2).

Depending on the immune context and the environmental stimuli, macrophages can adopt extreme phenotypes, ranging from pro-immunogenic TAM to tumorigenic TAMs [24, 25]. Indeed, our scRNAseq data of B16 tumors clearly revealed distinct TAM subsets (Fig. 6G). Remarkably, the vast majority of cells in the pro-immunogenic TAM cluster were derived from the Bisp-AFN group (96%), while most of the cells in the pro-tumorigenic TAM cluster belonged to the PBS group (94,5%). These data were visualized in an heatmap showing pooled clusters with TAM signatures. DE genes linked with pro-immunogenic TAMs are upregulated in the Bisp-AFN treatment, while DE genes specific for pro-tumorigenic TAMs are clearly upregulated in the PBS condition (Fig. 7B).

Together, these data clearly indicate the ability of Bisp-AFN treatment to reshape the suppressive tumor environment and revert the tumor-promoting activities of myeloid cells.

### Bispecific AcTaferon induces migration and potent maturation of dendritic cells

**Fig. 8:**
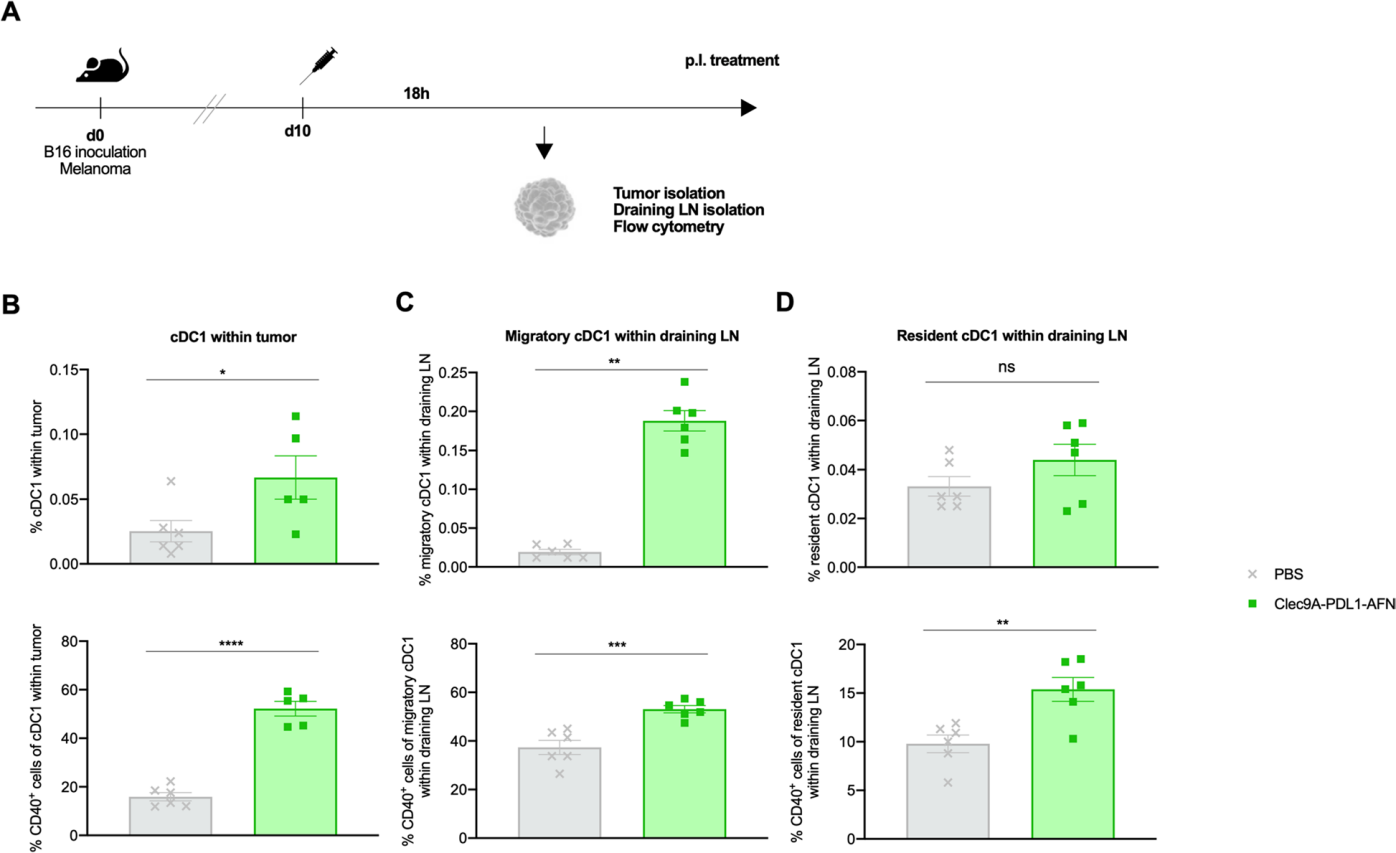
Bisp-AFN induces migration and potent maturation of dendritic cells. **A** Schematic representation of the experiment. Data obtained upon administration of PBS are represented in grey while data obtained after Clec9A-PDL1-AFN administration are visualized in green. **B**-**D** Presence and maturation status as determined by CD40 expression of cDC1 in B16 tumor (**B**) as well as migratory cDC1 (**C**) and resident cDC1 (**D**) in draining LN. cDCs were determined as CD45^+^ living cells, CD11c^+^MHC-II^+^. LN migratory cDCs are CD11c^intermediate^MHC-II^high^ while LN resident cDCs are CD11c^high^MHC-II^intermediate^. cDC1 were determined as CD11b^−^XCR1.^+^ cells within the described cDC population. Results show bar charts of individual values (**B**, **C**, **D**) with mean ± SEM. Graphs were analyzed using unpaired nonparametric Mann–Whitney t-test (migratory cDC1 within draining LN) or unpaired parametric t-test. * < 0.05; ** < 0.01; *** < 0.001; **** < 0.0001

cDC1 are considered as critical for antitumor immunity and their abundance within tumors has been associated with immune-mediated rejection and the success of immunotherapy [26]. We analyzed the presence and maturation status of cDC1 18 h after a single administration of either PBS or the Bisp-AFN (Fig. 8A, flow cytometry gating strategy in Suppl. Fig. 5). A significant increased presence of cDC1 in B16 tumors (Fig. 8B) as well as migratory cDC1 in draining LN (Fig. 8C), was observed upon administration of Bisp-AFN. Although not statistically significant, we observed a trend to increased presence of resident cDC1 in the draining lymph nodes (Fig. 8D) upon treatment with Bisp-AFN. In addition, these cDC1 showed a favorable matured phenotype demonstrated by their CD40 expression (Fig. 8B-D).

### Bispecific AcTaferon induces a shift from naive and dysfunctional T cells towards effector and reprogrammable CTLs

**Fig. 9:**
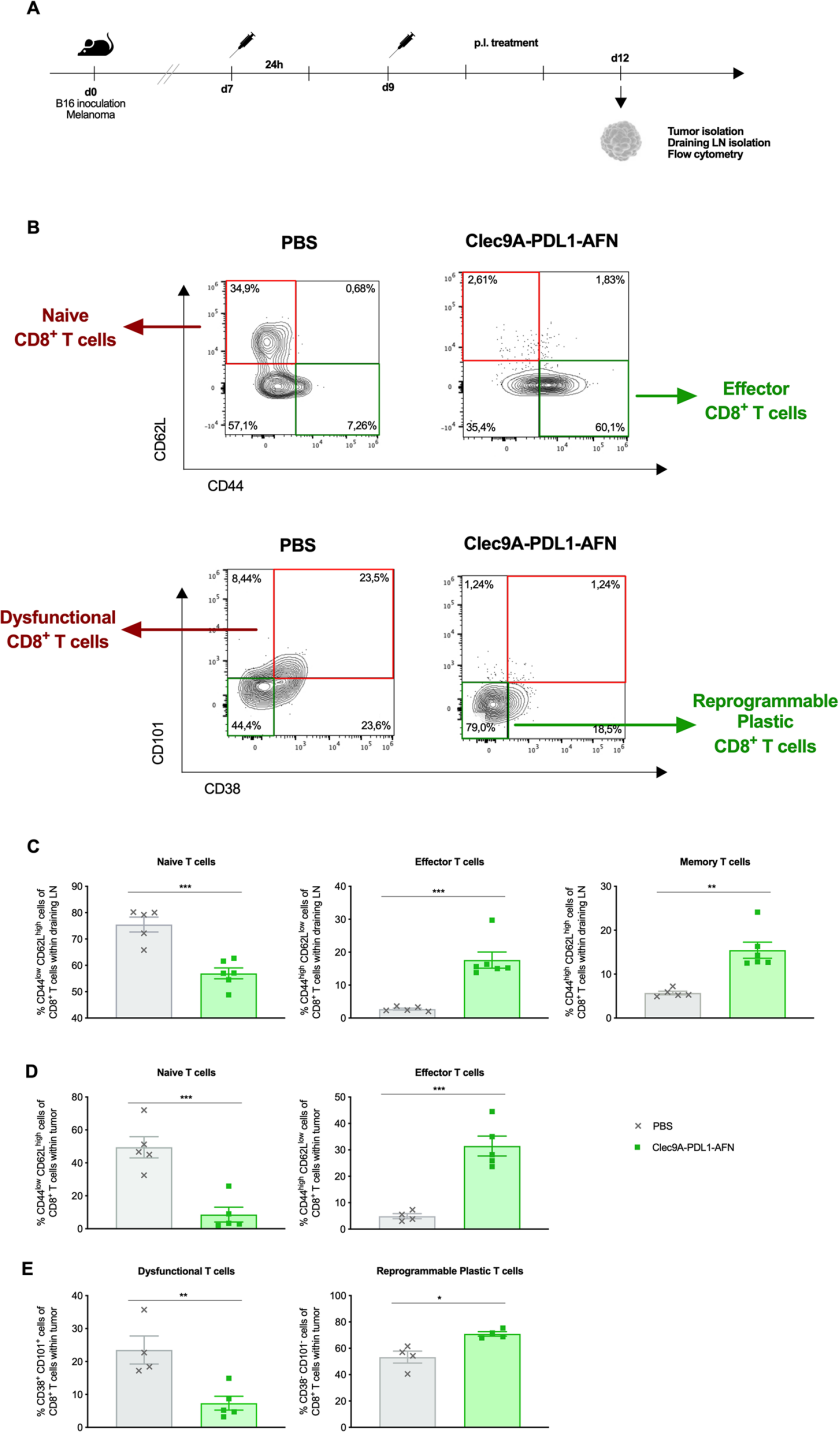
Bisp-AFN induces a shift from naive and dysfunctional T cells towards effector and reprogrammable CTLs. **A** Schematic representation of the experiment. **B** Flow cytometry gating strategy to determine CD8^+^ T cells: CD45^+^ living cells, TCR-β^+^ T cells, CD8^+^CD4^−^ T cells. Contour plots show the shift in expression of the indicated markers and cell populations over the different treatments. **C**, **D** Draining LN (**C**) and B16 tumors (**D**) were analyzed for CD44 and CD62L expression on CD8^+^ T cells. Naive cells were identified as CD44^low^CD62L^high^, effector T cells as CD44^high^CD62L^low^ and effector-memory T cells in draining LN as CD44^high^CD62L^high^. **E** CD8^+^ T cells in B16 tumors were analyzed for CD38 and CD101 expression with dysfunctional T cells described as CD38^+^CD101^+^ and reprogrammable, plastic T cells as CD38^−^CD101^−^. Results show bar charts of individual values with mean ± SEM. Data obtained upon administration of PBS are represented in grey while data obtained after Clec9A-PDL1-AFN administration are visualized in green. Shown is one representative experiment. Graphs were analyzed using unpaired parametric t-test (draining LN naive T cells, tumor effector T cells, tumor dysfunctional and reprogrammable T cells) or unpaired nonparametric Mann–Whitney t-test (draining LN effector T cells, draining LN memory T cells, tumor naive T cells). * < 0.05; ** < 0.01; *** < 0.001; **** < 0.0001

During cancer progression, CTLs often progress to a dysfunctional or exhausted state due to immune-related tolerance or immune-suppression within the tumor microenvironment [27, 28]. Therefore, we analyzed the status of CD8^+^ T cells in B16 tumors and draining LN (Fig. 9A-B). Administration of the Bisp-AFN drives T cells from a naive state into T cells with an effector phenotype in both draining LN (Fig. 9C) and tumor (Fig. 9B, D). In addition, in draining LN, CD8^+^ T cells with a memory phenotype, based on CD44^high^CD62L^high^ expression, could be increased after treatment with Bisp-AFN (Fig. 9C). Finally, in the PBS condition many tumor-resident CTLs showed a fixed dysfunctional state, while Bisp-AFN therapy resulted in presence of plastic, reprogrammable CTLs (Fig. 9B, E).

### Bispecific AcTaferon is a superior inducer of tumor-specific CTLs and promotes diversity in the TCR repertoire

**Fig. 10:**
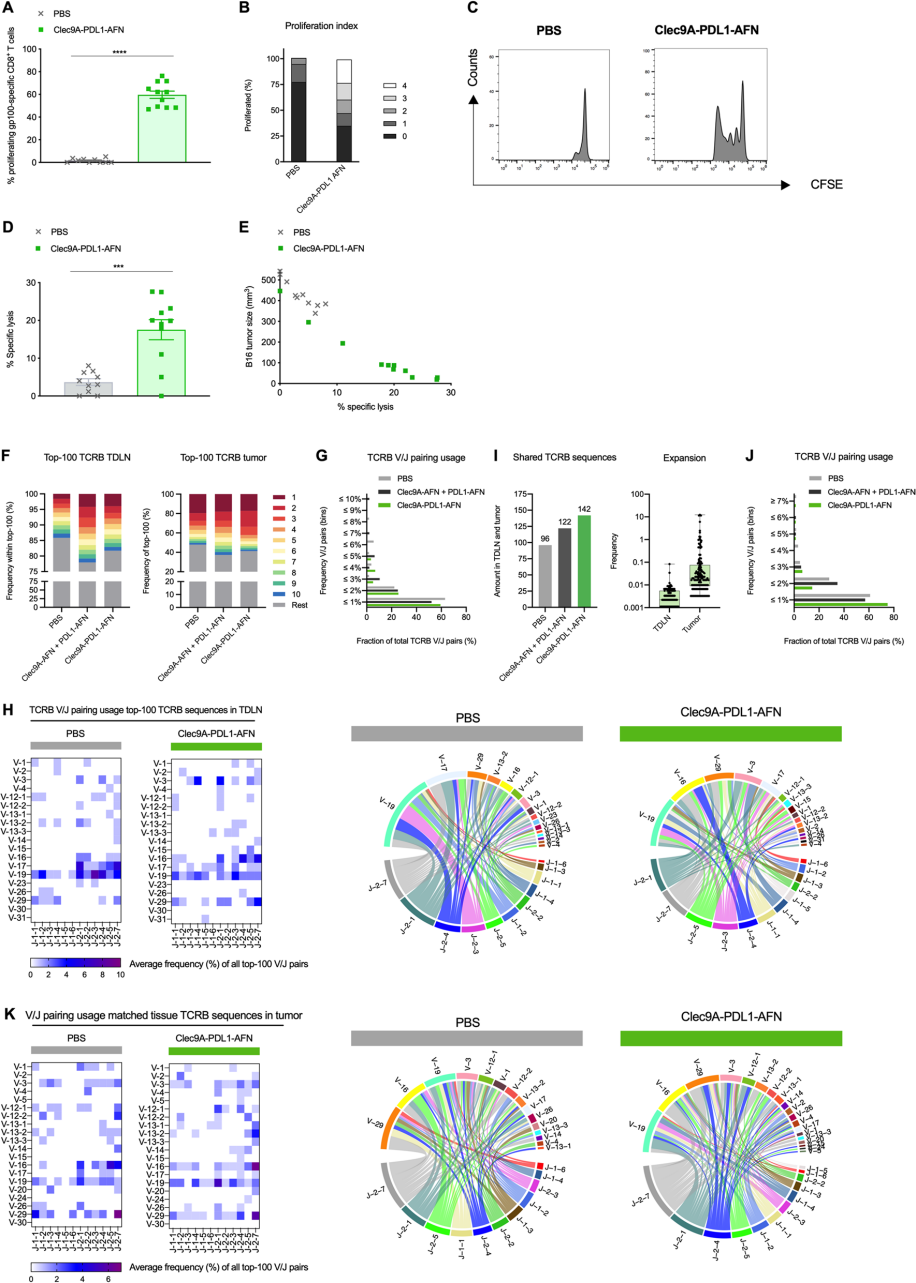
Bisp-AFN is superior in the induction of tumor-specific CTLs. **A**-**C** Flow cytometry analysis of gp100-specific CD8^+^ T cell proliferation in draining LN of B16-bearing mice p.l. injected with PBS (grey) or Bisp-AFN (green) at days 7 and 9 after tumor inoculation. One day prior to immunization, gp100-specific CD8^+^ T cells (pMel) were adoptively transferred in B16 tumor-bearing mice. Data show percentages of gp100-specific CD8^+^ T cells that have undergone at least one division (**A**), stacked bar chart representing the percentage of cells per division peak (**B**) and representative flow cytometry proliferation profile for each treatment (**C**). Shown is a summary of two independent experiments (6/group/experiment). **D**, **E** B16-bearing mice were p.l. injected at days 7 and 9 after tumor inoculation with the indicated conditions. Five days after the first delivery, the killing potency of the induced tumor-specific CD8^+^ T cells was analyzed in draining LN by in vivo cytotoxicity assay (**D**). The correlation between tumor size at time of read out and % specific lysis was plotted (**E**). A summary of two independent experiments is shown (5–6/group/experiment). **F** Frequencies of the top-10 largest T cell clones within the top-100 most-abundant TCRB sequences in the draining LN (*left*) and tumor (*right*). Note that colors do not represent the same sequence in different bars. **G**, **J** Frequency distribution plots of TCRB V/J pairing usage within the top-100 most-abundant LN sequences (**G**) and tumors sequences that also appear in the LN (**J**). **H**, **K** Average TCRB V/J pairing usage frequencies within the top-100 most-abundant LN sequences (**H**) and tumors sequences that also appear in the LN (**K**). Corresponding circos plots for each treatment group are shown next to the heatmaps. **I** Amount of TCRB sequences from the draining LN that are also retrieved in the matched tumor (*left*) with relative frequencies of these sequences after treatment with Bisp-AFN. A summary of three mice per group was analysed. Results show bar charts of individual values with mean ± SEM (**A**, **D**). An unpaired nonparametric Mann–Whitney t-test (**A**) or parametric t-test (**D**) was performed depending on Shapiro Wilk normality test * < 0.05; ** < 0.01; *** < 0.001; **** < 0.0001

To analyze the antigen-presentation skills of DCs, we performed a proliferation assay showing superior proliferation of gp100-specific T cells after Bisp-AFN treatment (Fig. 10A-C). Moreover, administration of Bisp-AFN resulted in a high fraction of gp100-specific T cells in the latest stages of proliferation (Fig. 10B-C). Induction of tumor-specific cytotoxic T cell responses is a fundamental objective in anticancer therapeutic strategies. Therefore, we analyzed the potency of the induced antigen-specific T cell response. Again, Bisp-AFN induced ample effects (Fig. 10D). In addition, the induced specific lysis inversely correlated with tumor size (Fig. 10E).

Next, we evaluated whether these DCs were able to establish diversity in the T cell repertoire, which we addressed via TCR analysis. To that end, B16 tumor-bearing mice were treated with either PBS or Bisp-AFN, after which CD8^+^ T cells were sorted from draining LNs and tumor for RNA sequencing. In both compartments, increased frequencies of several clones retrieved within the top-10 most-abundant TCRB sequences after treatment with the Bisp-AFN were noticed (Fig. 10F), which validates the findings from the abovementioned proliferation experiments (Fig. 10A-C). We further investigated this by measuring the average frequencies of TCRB V/J pairing usage per group in the LN. While CD8^+^ T cells isolated from control mice treated with PBS are more enriched in a selected number of relatively larger V/J pairings, the T cell response in Bisp-AFN-treated animals is spread over numerous V/J pairings that appear in lower frequencies, which is indicative of improved epitope spreading in the LN (Fig. 10G-H). Upon comparing matched tissues, we observed that more TCRB sequences initially retrieved in the draining LN also appeared and expanded in the tumor after treatment with Bisp-AFN, imposing a correlation between a durable antitumor response and T cell clone sharing between sentinel LN and tumor (Fig. 10I). In addition, average V/J pairing usage analysis for these particular shared clones reveals more oligoclonal CD8^+^ T cells expansion for certain TCRB V/J combinations in the tumor after treatment with Bisp-AFN compared with control (Fig. 10J-K).

Altogether, simultaneous targeting of type I interferon towards Clec9A and PD-L1 in a Bisp-AFN construct induces strong tumor-specific immune responses and increases diversity in the CD8^+^ T cell TCR repertoire without the need for tumor markers, as such representing a potent immunotherapeutic with broad applications.

## Discussion

Here, we described the superior efficacy of a bispecific AcTaferon construct simultaneously targeting type I IFN activity towards Clec9A- and PD-L1-expressing cells. This bispecific construct showed ample antitumor activity and resulted in modulation of the tumor microenvironment towards a pro-immunogenic state.

The general layout of the Bisp-AFN, by which two targeting moieties are linked together, resembles that of bispecific antibodies, engineered artificial antibodies capable of recognizing two epitopes of an antigen or recognizing two antigens [29], and in particular Bispecific T cell Engagers (BiTEs). The ‘BiTE-platform’ belongs to advanced T cell immunotherapeutic options of which CAR-T cells are most explored, and physically bridges two cells leading to the formation of an immunological synapse [30]. Given that the mode of action of Bisp-AFN invokes both cDC1 and PD-L1, including PD-L1 expressed on the tumor cells, tethering and retention may possibly also lead to formation of a ‘synapse-like event’ between the immune cell and the tumor cell. This is an interesting notion that might have possibly contributed to the observed effects and is currently subject of further investigation. Type I IFN signaling is crucial as unarmed bispecific constructs are completely inactive, suggesting that pure tethering alone is insufficient. In addition, co-localized signaling and co-signaling loops might be in play. It is known that type I IFNs induce upregulation of PD-L1 resulting in expression of PD-L1 on tumor cells and immune cells [11, 31, 32], making them more susceptible to IFN activity by PD-L1-targeted AFN, as such creating a feed-forward loop. Noteworthy, our scRNAseq data revealed a significant expression of PD-L1 on subpopulations of myeloid cells including TANs and TAMs expressing pro-immunogenic phenotype as well as moDCs, which correlated with the increased expression of genes related with IFN activity. One of the main signaling roles of the PD-L1 molecule includes protection of the tumor cells from the cytolytic effects of type I and type II interferons [33]. However, several studies raised the debate on the importance of PD-L1 expression on tumor cells versus host immune cells for successful antitumor therapies [34–37]. Here, we show that PD-L1 expression on tumor cells was partially responsible for the antitumor efficacy induced by the treatment with the Bisp-AFN. Of note, also proliferating NK and NKT cells are significantly increased in the tumor upon Bisp-AFN treatment, suggesting a co-signaling loop for NK cells and cDC1. Although not in the scope of this paper, this might be an interesting observation to further explore in view of the findings by Barry and colleagues [18] showing correlation of NK cell frequency in human cancers with protective intratumoral stimulatory DCs, increased responsiveness to immune checkpoint therapy and hence increased overall survival.

Our scRNAseq data showed decreased presence of cDC1 in B16 tumors three days after the first AFN administration, which was explained by their increased motility towards draining LN. This favors the induced tumor-specific immunity, as DC migration directly correlates with the extent of T cell proliferation and effector cell differentiation [38]. On top, few hours after Bisp-AFN delivery, increased presence of cDC1 in B16 tumors and draining LN together with a favorable maturation pattern was observed, in sharp contrast to administration of PBS. These is important as cDC1 are regarded as major players in regulating anticancer immune responses locally within tumor tissue [39], as they attract T cells [40], re-stimulate and expand tumor-specific CD8^+^ T cells [41] and support T cell effector function [42]. Furthermore, we could demonstrate the presence of infl-cDC2 in a tumor setting. In this regard, Bosteels et al*.* described that upon inflammation cDC2 acquire a hybrid inf-cDC2 phenotype capable of optimally priming both CD4^+^ and CD8^+^ T cell immunity [22]. As this differentiation was driven by type I IFN [22], we hypothesize an important role for AFN-signaling in the induction of inf-cDC2 detected in our study. Indeed, scRNAseq showed only few inf-cDC2 in the PBS condition compared to abundant presence in the Bisp-AFN condition.

In addition, our results reveal the modulation of several suppressive immune cell types in the tumor niche. Indeed, our scRNAseq data showed increased presence of pro-immunogenic TAMs as well as TANs. It has been extensively reported that depending on the environmental stimuli, TAMs can adopt distinct and opposing functions during differentiation [25, 43–45]. Our results indicate that the Bisp-AFN can provide the right stimuli to direct cells towards this favorable profile. One other strategy to improve the therapeutic efficacy against tumors includes reversing T cell suppression and tolerance. Tregs are regarded as key players in maintaining immunological tolerance and immunosuppressive effects, thereby limiting the power of cancer immunotherapies [46, 47]. Typically, their increased presence in tumors has been correlated with poor survival [48, 49]. In this regard, scRNAseq analysis revealed a decreased proportion of Tregs in B16 tumors upon treatment with the Bisp-AFN. Furthermore, we report on the induction of effector CD8^+^ T cells with a plastic phenotype upon treatment with the Bisp-AFN. Again, this stands in sharp contrast to the mainly naive and/or dysfunctional CD8^+^ T cell phenotype observed after PBS treatment. These results were observed already five days after the first AFN delivery and three days after the second administration, which might be considered as “early” in T cell differentiation and reprogramming. However, T cell dysfunction as observed in late stage clinical cancer patients may have already been established early during tumorigenesis [50]. Our results show that Bisp-AFN drives CD8^+^ T cell expansion, acquisition of an effector phenotype and effector functionality, next to increased diversity in the CTL TCR repertoire. TCR analysis revealed epitope spreading in the draining LN, evidenced by distribution of the T cell clonotypes over multiple TCRB V/J combinations present in lower frequencies, which indicates a highly polyclonal response. In addition, we showed a higher overlap in T cell clones between the sentinel LN and the tumor, together with a more pronounced oligoclonal expansion of several of these clones in tumors of mice treated with Bisp-AFN. Although this warrants further investigation, one might argue that these findings refer to better trafficking of the induced antigen-specific T cells from the LN towards the tumor area and/or the existence of tertiary lymphoid structures. Altogether, Bisp-AFN is a generic strategy that enabled very potent tumor-specific cytotoxic T lymphocyte responses and antitumor activity. In combination with a non-curative dose of chemotherapy, we showed tumor clearance and immunological memory, therapeutic immunity on large established tumors and blunted tumor growth at distant sites.

In conclusion, treatment with the Bisp-AFN fulfills two key aspects of cancer immunotherapy by promoting antitumor immunity and overcoming tumor-induced immune suppression. Indeed, our Bispecific AcTaferon affects many cells that play an important role in cancer immunoediting and is highly efficient in reshaping the suppressive tumor microenvironment. Therefore, this strategy might be considered as a safe, potent and broad-spectrum therapeutic approach to solid tumors and hematological cancers.

## Materials and methods

### Construction and production of the AcTakines

The mutations Q124R or R149A were introduced into the human IFNα2 sequence by site-directed mutagenesis using the QuikChange II-E Site-Directed Mutagenesis Kit (Agilent Technologies). Single domain antibodies (sdAb) were generated at the VIB Nanobody Core, as described previously [9]. The mutated cytokines as well as the wt human IFNα2 (not active in mice) and wt murine IFN were coupled N-terminally to targeting sdAbs via a 20xGGS-linker. For bispecific AcTakines, two different targeting moieties were connected with a 10xGGS linker and coupled via a 20xGGS linker to the mutated cytokine. AcTakines were constructed and produced as previously described [10].

### Mice

Female, 7–8 weeks old C57BL/6 J and 7–8 weeks old (s.c. tumor inoculation) or 10–12 weeks old (orthotopic tumor inoculation) Balb/cJ mice were purchased from Charles River Laboratories (France). pMel mice that carry a transgenic TCR specific for the MHC-I restricted gp100 peptide were a kind gift from Prof. K. Breckpot (VUB, Belgium). All strains were bred in our own facility. NOD-*scid* IL2Rγ^null^ (NSG) mice were bred at our own facility or at the breeding facility of the University Hospital Ghent (UZGhent, Belgium). Humanized Immune System (HIS) mice were generated as previously described [10]. Mice were housed in individually ventilated cages under pathogen-free conditions in a temperature- and humidity-controlled environment with 12/12 h light/dark cycle and received food and water ad libitum.

Animals were treated according to the Federation of European Laboratory Animal Science Association (FELASA) guidelines. Experiments were reviewed and approved by the Ethical Committee of Ghent University (ECD15/88, ECD18/82 and ECD17/11). Mice were allocated randomly to a group. Where possible, the investigators were blinded during data collection and analysis.

### Cell lines

Murine tumor cell lines include melanoma cell lines B16, B16-PD-L1^−/−^ and B16-mCD20-IFNAR^−/−^ as well as 4T1 mammary carcinoma. For the humanized model, human follicular B cell lymphoma RL cells were used. B16, 4T1 and RL cell lines were purchased from American Type Culture Collection (ATCC) and cultured in conditions specified by the manufacturer. B16-mCD20-IFNAR^−/−^ cells were generated as previously described [11]. The B16-mCD20-PD-L1^−/−^ cell line was generated via the CRISPR-Cas9 editing system, using a gRNA sequence targeting exon 3 of PD-L1, 5’- AGGTCCAGCTCCCGTTCTAC-3’ (determined via http://crispr.mit.edu). The gRNA was cloned in the pSpCas9(BB)-2A-Puro vector (PX459) [51] and transfected into B16 cells via Jetprime. After 4 weeks of culture with 1 µg/ml puromycine, negative selection was performed using MACS with anti-CD274-PE (eBioScience) and anti-PE microbeads (Miltenyi Biotec). The absence of PD-L1 was verified with flow cytometry. All cell lines used for inoculation were free of mycoplasma. Cell lines were analyzed by Eurofins Scientific (Luxembourg) for authentication.

### Tumor implantation, analysis and treatments

For s.c. tumor models, cells were injected in 50 μl PBS suspension and include 6 × 10^5^ B16, B16-mCD20-IFNAR^−/−^, B16-PD-L1^−/−^ cells or 10^5^ 4T1 cells. For the orthotopic 4T1 tumor model, mice were anesthetized with a mixture of ketamine (Nimatek, 70 mg/kg, EuroVet) and xylazine (Rompun, 10 mg/kg, Bayer), whereupon the fourth mammary fat pad was surgically exposed and injected with 10^4^ 4T1 cells in a volume of 10 μl PBS. The incision was closed using 6–0 coated vicryl absorbable suture (Ethicon). For the humanized mice model, HIS mice were s.c. inoculated with 2 × 10^6^ human follicular lymphoma RL cells, 12 weeks after human stem cell transfer. In addition, mice received daily intraperitoneal (i.p.) injections for 8 to 12 times with 30 μg Fms-like Tyrosine kinase 3 ligand (Flt3L) protein, known to activate hematopoietic progenitors and playing an important role in the development and mobilization of DCs [52, 53], according a schedule indicated in the experiment layout.

Tumor size was defined by caliper measurements of tumor dimensions in mm and calculated using the formula for a prolate ellipsoid *i.e.* length x width^2^/2. Survival was indicated as tumors < 1000 mm^3^. To analyze tumor immunity, mice were re-challenged on the contralateral flank with a new dose of tumor cells. Tumor-free mice in the orthotopic setting were re-challenged subcutaneously.

To analyze abscopal effects, a 2-site tumor model was performed. To that end, mice were inoculated with a primary tumor at the right flank followed by a secondary tumor at the left flank, three days later. When both tumors were palpable, only the primary tumor (right flank) was treated after which systemic effects of the local treatment were evaluated on both the treated as well as the non-treated, distant tumor.

Tumor treatments were done perilesionally (p.l.), which is s.c. at the tumor border. For tumor growth experiments, daily treatments were performed for at least 8 to 10 times, indicated by the black line in the X-axis. For scRNAseq, B16 tumors were p.l. injected on three consecutive days and isolated 6 h after the last injection. For analysis of DC presence and maturation, mice received a single p.l. injection. For analysis of CTL influx, activation and tumor-specificity, B16 tumors were injected twice, with a one-day interval. For TCR analysis, daily treatments were performed for 6 consecutive days.

As a control, mice were injected with PBS. For the different treatment groups, protein dose was kept constant to finally obtain equal targeting amongst the different groups. The different treatment groups include 30 μg of the Bisp-AFN Clec9A-PDL1-AFN, 30 μg Clec9A-PDL1-IFN or Clec9A-PDL1-huIFN. For combination therapies, a non-curative dose of doxo (3 mg/kg) was injected s.c. in near proximity of the tumor every other day for 5–6 times as indicated by the red arrows.

### Hematological analysis

One day after the last treatment, blood was collected using EDTA-coated microvette tubes (Sarstedt) and analyzed for blood parameters in a Hemavet 950FS whole blood counter (Drew Scientific).

### Single cell RNA sequencing

To minimize bias effects and to finally obtain a sufficient number of immune cells, tumors of six individual mice for each treatment condition were pooled. Approximately 4 × 10^4^ CD45^+^ live immune cells were sorted from single cell suspensions of B16 tumors using BD FACS Aria™ III cell sorter (Becton Dickinson). Sorted cells were re-suspended at an estimated final concentration of 1000 cells/μl in PBS/0,04% BSA to proceed to single cell RNA sequencing.

To that end, the cellular suspensions were loaded on a GemCode Single Cell Instrument (10 × Genomics) to generate single cell Gel Bead-in-Emulsions (GEMs). Single cell RNA-seq libraries were prepared using GemCode Single-cell 3’Gel Bead and Library Kit (10 × Genomics) according to the manufacturer’s instructions. GEM-RT was performed (45 min at 55 °C, 5 min at 85 °C, end at 4 °C) in 96-deep well reaction modules followed by break down of the GEMs and clean-up of the cDNA using DynaBeads MyOne Silane Beads (Thermo Fisher Scientific) and SPRIselect Reagent Kit (Beckman Coulter). Next, cDNA was amplified with 96-deep well reaction module according to following schedule: 3 min at 98 °C followed by 12 cycle times of 15 s at 98 °C, 20 s at 67 °C, 1 min at 72 °C. Last step included 1 min at 72 °C and end at 4 °C. The amplified cDNA product was cleaned up using SPRIselect Reagent Kit prior to enzymatic fragmentation. GemCode Single Cell 3’ Library kit (V2 chemistry) was used to generate indexed sequencing libraries and include following intermediates: end repair, A-tailing, adaptor ligation, post-ligation SPRIselect cleanup and sample index PCR. Pre-fragmentation and post-sample index PCR samples were analyzed using Agilent 2100 Bioanalyzer.

Sequencing was performed at VIB Nucleomics Core. To that end, sequencing libraries were loaded on a HiSeq4000 with sequencing settings following recommendations of 10xGenomics. 10x’s CellRanger software was used for demultiplexing of the raw data, whereupon these reads were used as the input for CellRanger (10X Genomics), which align the reads to the mouse reference genome (mm10) using STAR and collapse to unique molecular identifier (UMI) counts. Scran and scatter R package was used for processing of the data in accordance to the workflow as described [54, 55]. Outlier cells were identified based on three metrics *i.e.* library size, number of expressed genes and mitochondrial proportion. Cells from the control (PBS treated) sample were tagged as outliers when they were three median absolute deviations (MADs) away from the median value of library size and number of expressed genes, four MADs for the sample treated with Clec9A-PDL1-AFN. Likewise for both samples, cells 25 MADs away from the median proportion were identified as outlier. These outlier cells were removed from the analysis. Genes expressed in less than 3 cells and cells expressing less than 200 genes were removed. The samples were aggregated using the merge function, counts were normalized and log2 transformed using the NormalizeData function, both from the Seurat R package (v4.0.2) using default parameters. Detecting highly variable genes, scaling, finding clusters, and creating UMAP plots was done using the Seurat pipeline. Clustering was performed using the first 37 principal components and a resolution of 1.5.

### In vivo proliferation assay

Gp100-specific CD8^+^ T cells were isolated from spleens of naive pMel mice using MACS separation protocols (Miltenyi Biotec) and subsequently labeled with 0,5 μM carboxyfluorescein succinimidyl ester (CFSE, Life Technologies). Cells were adoptively transferred into B16-tumor bearing mice. Mice were p.l. injected with PBS or the AcTakine condition twice, one day after adoptive transfer and an additional administration 2 days later. Proliferation of gp100-specific T cells, measured as a dilution of CFSE, was analyzed in draining LN three days after the last AcTakine delivery. Samples were acquired on an Attune Nxt Acoustic Focusing Cytometer (Life Technologies) and analyzed using FlowJo software.

### In vivo cytotoxicity assay

To analyze the potency of the induced antigen-specific T cells, an in vivo cytotoxicity assay was performed. To that end, B16 tumor-bearing mice were p.l. injected twice with a one-day interval with PBS or the Bisp-AFN. Three days after the last delivery, the killing assay was performed according to current Protocols of Immunology [56]. In brief, spleen cells from syngeneic mice were pulsed or not with 5 μM EGSRNQDWL peptide (mouse gp100 25–33). Peptide pulsed cells were labeled with 2,5 μM CFSE after which they were mixed at a 1:1 ratio with 0,25 μM CFSE labeled, non-peptide pulsed cells. At least 10^7^ cells were injected intravenously (i.v.). Spleen and draining LN were isolated 24 h later. Specific lysis was analyzed in draining LN using an Attune Nxt Acoustic Focusing Cytometer (Life Technologies) to acquire the samples and FlowJo software to analyze the data. The percentage killing was calculated as described [56].

### CD8^+^ T cell sorting, RNA isolation and TCR repertoire analysis

Daily perilesional treatments were performed for 6 consecutive days after which CD8^+^ T cells were sorted from draining LNs and B16 tumor tissue using a three-laser FACSAria II (BD Biosciences) and captured in 350 μL RLT lysis buffer supplemented with β-mercaptoethanol. RNA was isolated using the RNeasy Plus Micro Kit (74034, Qiagen) according to the manufacturer’s instructions. Concentration, quality and integrity of the RNA was measured using an Agilent 2100 Bioanalyzer (Agilent). Samples for which RIN ≥ 8 and 280/260 and 260/230 values > 1.8 were used for library preparation with the Qiagen QIAseq™ Mouse TCR Panel Immune Repertoire RNA Library Kit (Qiagen) following the manufacturer’s instruction. RNA was first reverse-transcribed into cDNA using primers specific to the TCR region, after which double-stranded cDNA was generated, end-repaired and A-tailed. All original cDNA molecules were ligated to a 12-base random adaptor sequence used as a Unique Molecular Identifier (UMI). Enrichment for target sequences was performed with a first PCR reaction using a primer against the TCR constant region and a primer against the adaptor sequence. A second PCR was used to further amplify the library, add platform-specific adaptor sequences and introduce additional sample indices. Samples were next pooled and sequenced in two Illumina MiSeq v2 500 runs. All raw read data was merged and analyzed using the CLC Genomics Workbench (GWB) 21 (https://digitalinsights.qiagen.com/) [57] via the mouse “Immune Repertoire Analysis” workflow. We used default parameters for this analysis, except for “min UMI group size” (set to 1 to increase sensitivity).

### Flow cytometry

Commercially available antibodies used throughout the paper are listed in Supplementary Table 2. To prevent aspecific binding, cells were pre-incubated with anti-mouse CD16/CD32 (clone 93, eBioScience). Samples were acquired on an Attune Nxt Acoustic Focusing Cytometer (Life Technologies) and analyzed using FlowJo software.

### Statistical analysis

Shapiro–Wilk Normality test was performed to determine Gaussian distribution (α = 0,05) of the data. When data were normally distributed according to Shapiro–Wilk testing, unpaired two-tailed student t-test or ANOVA followed by Tukey’s multiple comparisons test was performed. If data were not normally distributed according to Shapiro–Wilk testing, unpaired nonparametric Mann–Whitney test or ANOVA Kruskal–Wallis test with Dunn’s multiple comparisons test was performed. Time to reach a specific tumor size as well as survival were represented in a Kaplan Meier plot compared by log-rank (Mantel-Cox) testing. Statistical analyses were performed using the GraphPad Prism software. The numbers of independent biological replicates or the numbers of individual mice have been indicated in the figure legends.

### Supplementary Information

**Additional file 1. **

## Data Availability

All data supporting the findings of this study are available within the article of upon further request.

## Abbreviations

AFN: AcTaferon
APC: Antigen-presenting cell
Bisp-AFN: Bispecific AcTaferon
BiTE: Bispecific T cell Engager
CAR: Chimeric antigen receptor
CD: Cluster of differentiation
cDC: Conventional Dendritic cell
CFSE: Carboxyfluorescein succinimidyl ester
CLEC9A: C-type lectin domain containing 9A
CTL: Cytotoxic T Lymphocyte
DC: Dendritic cell
DE: Differentially expressed
doxo: Doxorubicin
Flt3L: FMS-like Tyrosine kinase 3 Ligand
GEM: Gel Bead-in-Emulsions
HIS: Humanized Immune System
IFN: Interferon
IFNAR: IFN-α and -β Receptor
Inf-cDC2: Inflammatory cDC2
i.p.: Intraperitoneal
LN: Lymph node
MAD: Median absolute deviation
migrDC: Migratory DC
moDC: Monocyte-derived dendritic cell
NK: Natural Killer
NKT: Natural Killer T cell
PD-1: Programmed cell Death protein 1
pDC: Plasmacytoid DC
PD-L1: Programmed Death-Ligand 1
p.l.: Perilesional
s.c.: Subcutaneous
scRNAseq: Single cell RNA sequencing
sdAb: Single domain Antibody
SEM: Standard error of the mean
TAM: Tumor-associated macrophages
TAN: Tumor-associated neutrophils
TCR: T cell receptor
Treg: Regulatory T cell
UMAP: Uniform Manifold Approximation and Projection
UMI: Unique molecular identifier
wt: Wild type
