# Supplementary material for: A bispecific Clec9A-PD-L1 targeted type I interferon profoundly reshapes the tumor microenvironment towards an antitumor state
Source: Mol Cancer. 2023 Nov 29;22:191. doi: 10.1186/s12943-023-01908-6 (PMC10685570; doi:10.1186/s12943-023-01908-6)
Supplement: Supplementary file 1 — Additional file 1. [file 12943_2023_1908_MOESM1_ESM.docx]

**Supplementary information**


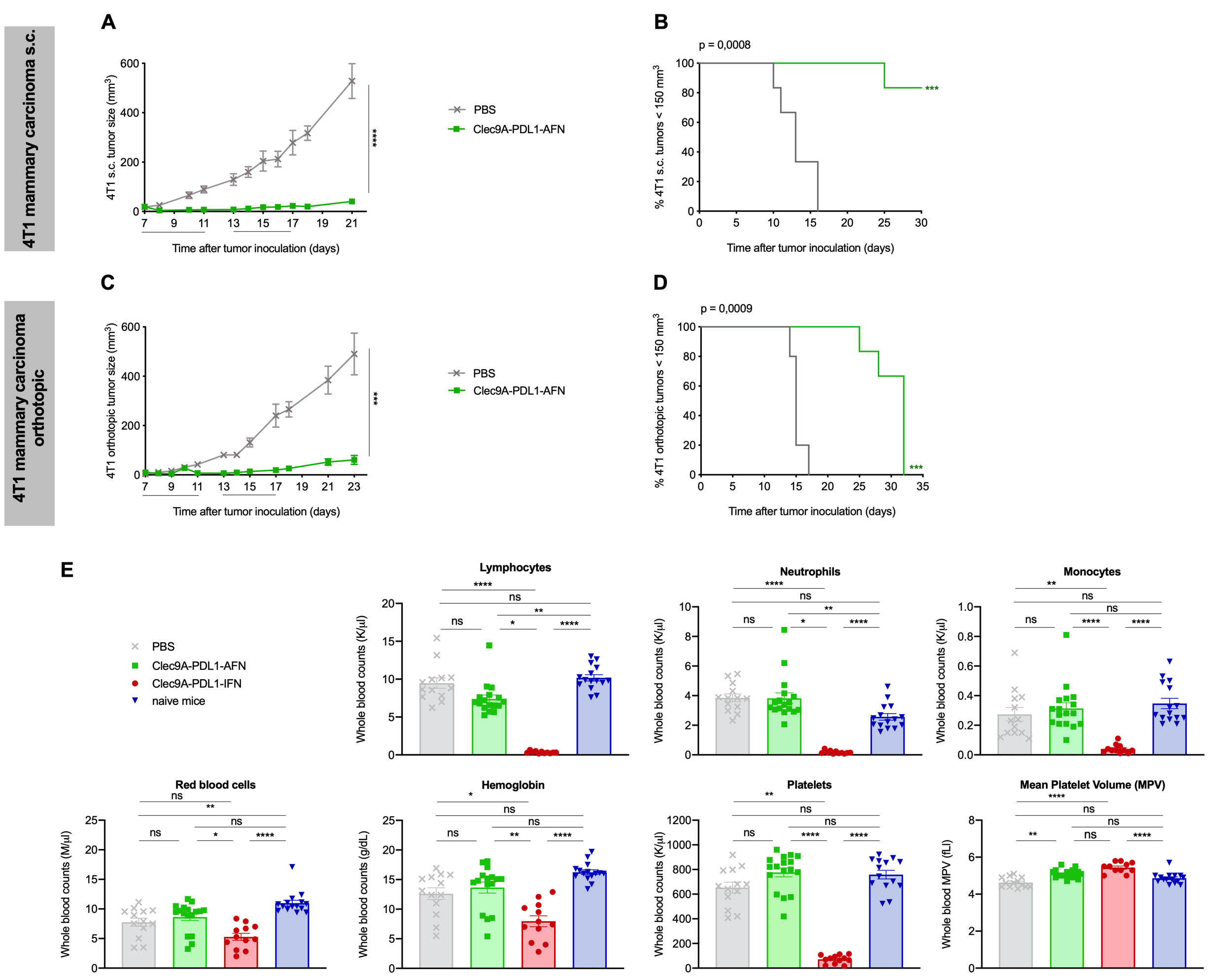


**Supplementary Figure 1.** Superior antitumor potential and absence of toxic side effects. **A-B.** 4T1 mammary carcinoma cells were inoculated s.c. (n=6). **C-D.** Orthotopic 4T1 mammary carcinoma model. Shown is one representative experiment out of three (6/group/experiment). Figures show tumor growth (A, C) as well as time to reach a 4T1 tumor volume of 150 mm^3^ (B, D). **E**. B16 cells were s.c. inoculated in the flank of C57BL/6 mice. Tumors were p.l. treated ten times with PBS (grey), 30 μg of wild type IFN (red), the Bispecific-AFN (green). Naive mice (blue) were included as a control. Hematological analysis (Hemavet) of blood parameters was performed one day after the last p.l. administration. Graphs show a summary of individual values ± SEM of 3 independent experiments (6 mice/group/experiment).

Tumor growth (A, C) was analyzed at day 21 (A) or d23 (C) respectively, using unpaired two-tailed student t-test. Black lines underneath the X-axis depict the treatment time. Time to reach a specific tumor size (B, D) was represented in a Kaplan Meier plot compared by log-rank (Mantel-Cox) test. Bar plots (E) were analyzed using One-way ANOVA Kruskal-Wallis with Dunn’s multiple comparisons test. * < 0.05; ** < 0.01; *** < 0.001; **** < 0.0001.


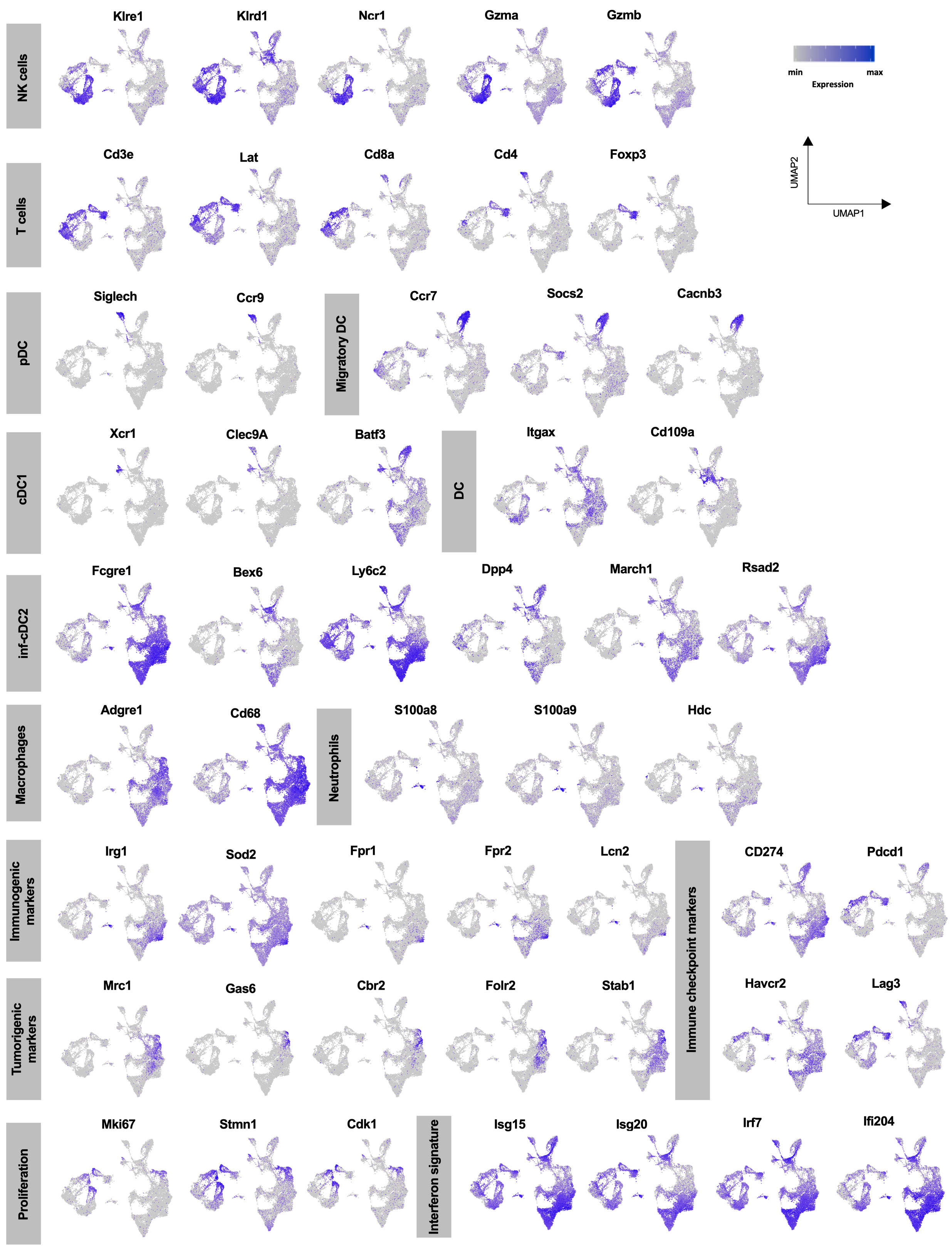


**Supplementary Figure 2**. Annotation of scRNAseq data of B16 tumors. UMAP representation showing feature plots of key DE- genes to annotate the different clusters.


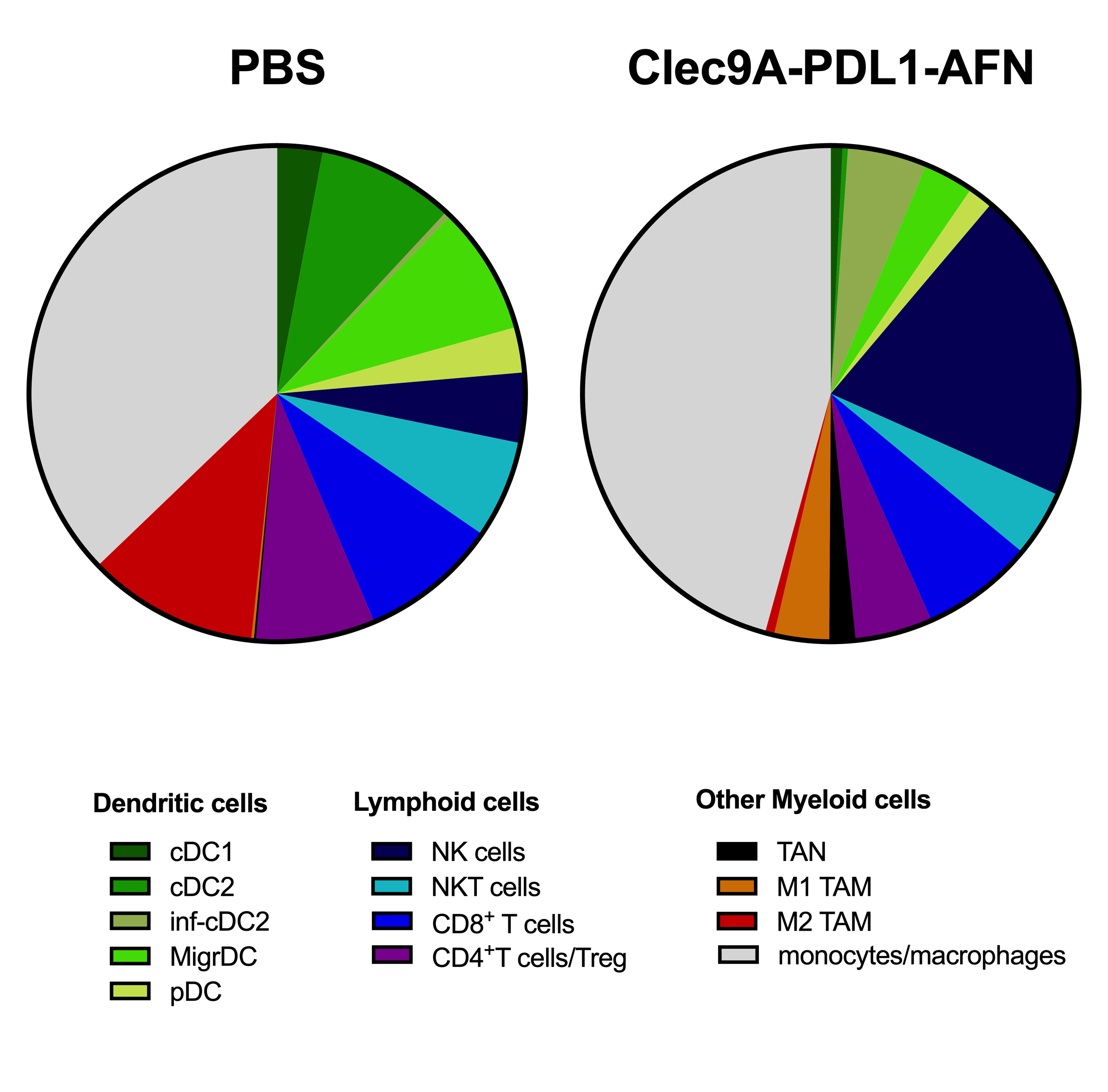


**Supplementary Figure 3.** scRNAseq of B16 tumors revealing B16 immune cell composition. Parts of whole graphs showing the relative immune cell composition of the B16 tumor over the different treatments.


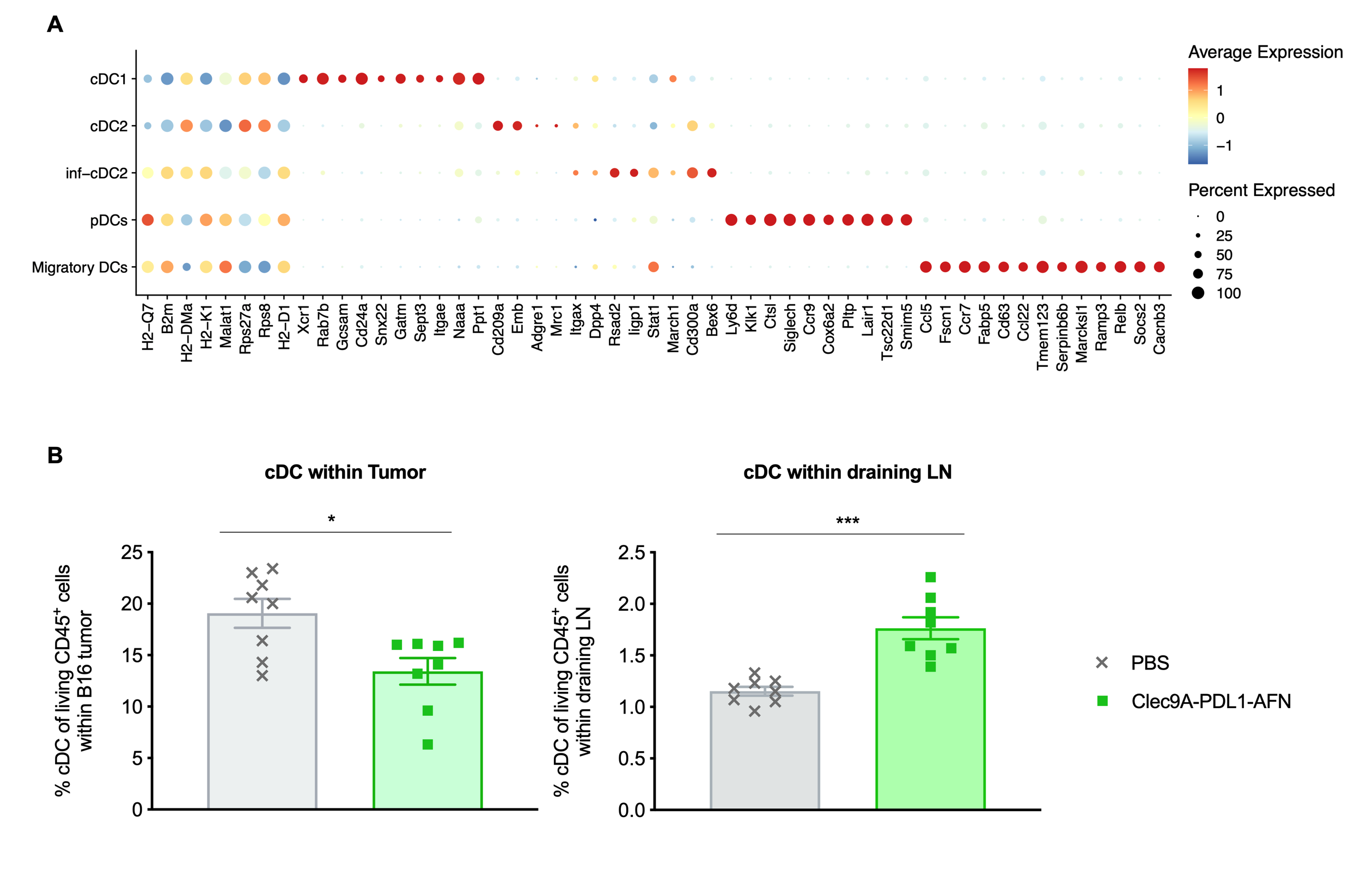


**Supplementary Figure 4.** Analysis of dendritic cells. **A**. scRNA sequencing annotation plot showing differentially expressed (DE) genes in the X-axis to determine the different DC populations (Y-axis). The size of the dot indicates the number of cells that express the gene of interest. The color intensity reflects the expression level (red = high, blue = low). **B**. Flow cytometer analysis of DCs in B16 tumor and draining lymph nodes (LN) according to the scRNAseq experimental set-up. B16 tumors were treated three times p.l. with PBS (grey) or Bisp-AFN (green), every day. Six hours after the last administration, tumors and draining LN were isolated and analyzed using flow cytometry. cDCs were determined as CD45^+^ living cells, CD11c^+^MHC-II^+^. Results show bar charts of individual values (B) with mean ± SEM. Shapiro Wilk normality test was performed. Graphs were analysed using an unpaired nonparametric Mann-Whitney t-test * < 0.05; ** < 0.01; *** < 0.001; **** < 0.0001.


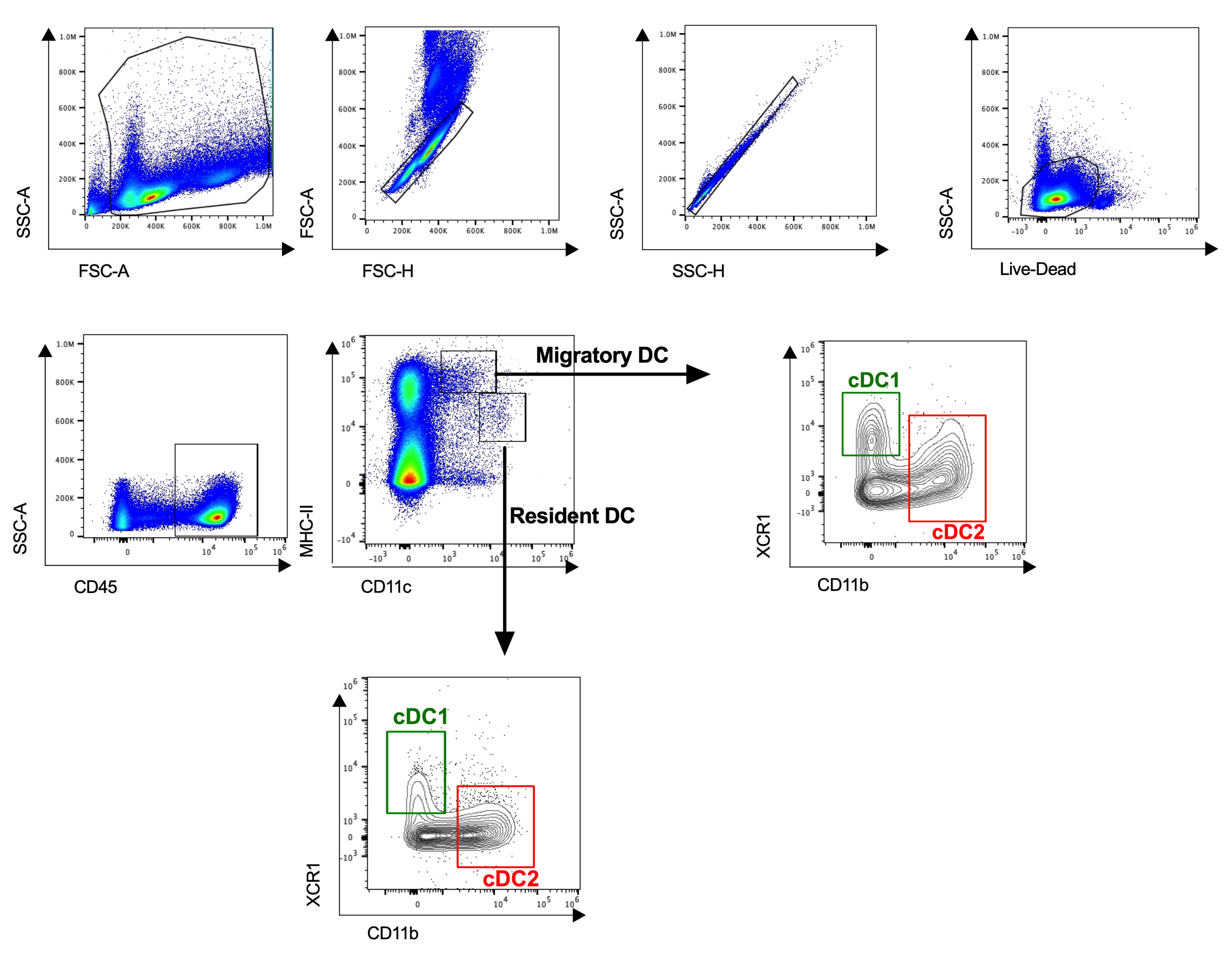


**Supplementary Figure 5**. Flow cytometry gating strategy to determine cDC in draining lymph nodes. cDCs were determined as CD45^+^ living cells, CD11c^+^MHC-II^+^. LN migratory cDCs are CD11c^intermediate^MHC-II^high^, while LN resident cDCs are CD11c^high^MHC-II^intermediate^. cDC1 are determined as CD11b^-^XCR1^+^ cells within the described cDC population, while cDC2 are CD11b^+^XCR1^-^.

**Supplementary Table 1**. scRNAseq marker genes that were used to identify immune cell clusters within the B16 tumor.

| **Cell Type** | **Markers** |
| --- | --- |
| **Antigen-presenting cells (APC)** | |
| General APC | H2DMb2, H2Eb1, H2Aa, H2Dma, H2Ab1, H2Oa, Cd300a |
| cDC1 | Xcr1, Irf8, Batf3, Id2, Gcsam, Itgax |
| cDC2 | Itgam, Sirpa, Itgax, Relb, Irf4, Zeb2, Tnni2, CD209a, Mafb^-^ |
| Inf-cDC2 | Fcgre1, Mafb, Bex6, Ly6c  Dpp4, Irf8, Sirpa, March1  Rsad2, Iigp1, Stat1, Ifit1, Ifit3, Ifi205 |
| Migratory DC | Ccr7, Socs2, Cacnb3, Adcy6, Tmem123 |
| pDC | Siglech, Ccr9, Pacsin1, Tcf4, Bst2 |
| **Lymphoid cells** | |
| NK cells | Gzma, Gzmb, Nkg7, Klre1, Ncr1, Klrd1 |
| NKT cells | Cd3e, Lat, Ncr1, Klre1, Klrd1, Ltb, Cd3d, Cd3g |
| General T cells | Thy1, Lat, Ms4ab4b, Cd3d, Cd3g, Cd3e |
| CD8 T cells | Thy1, Lat, Cd3e, Cd8a |
| CD4 T cells | Thy1, Lat, Cd3e, Cd4 |
| Tregs | Thy1, Lat, Cd3e, Cd4, Il2ra, Foxp3 |
| **Other myeloid cells** | |
| Monocytes/Macrophages | Fcgr1, Adgre1, Fcgr3, Fcgr4, Cd68 |
| Non-classical monocytes | Ace, Eno3, Adgre4, Csf1r, Cxc3cr1 |
| Classical Monocytes | Chil3, Ly6c2, Ccr2, Ier3, |
| M1 TAM | Fcgr1, Adgre1, Cd38, Fpr2, Nos2, Sod2, Irg1, Ly6c2 |
| M2 TAM | Fcgr1, Adgre1, Mrc1, Arg1, Gas6, Cbr2, Folr2, Stab1 |
| Neutrophils | S100a8, S100a9, Retnlg, Wfdc21, Lcn2, Ngp, Hdc, Lrg1 |
| **Other** | |
| Proliferation | Mki67, Stmn1, Esco2, Ccnb2, Cdk1 |
| IFN signature | Isg15, Isg20, Ifit3, Ifit1, Ifit2, Ifi44L, Ifi27, Ifitm1, Ifitm2, Mx1, Cxcl9, Cxcl10, Cxcl11, Socs1, Ccl8, |
| Redox genes | Gsr, Mif, Gpx1, Ier3, Cx3cr1, Mgst1 |

**Supplementary Table 2**. Antibodies used for flow cytometry.

| **Marker** | **Fluorochrome** | **Clone** | **Source** |
| --- | --- | --- | --- |
| CD16/CD32 | purified | 93 | eBioScience |
| Live Dead | Fixable Aqua |  | ThermoFisher Scientific |
| CD45 | APC-Cy7 | 30-F11 | Biolegend |
| CD3 | Alexa Fluor700 | 17A2 | eBioScience |
| CD19 | Alexa Fluor700 | eBio1D3 | eBioScience |
| CD11c | PE-Cy7 | N418 | Biolegend |
| CD11c | APC | N418 | Biolegend |
| MHCII | BV711 | M5/114.15.2 | Biolegend |
| MHCII | FITC | M5/114.15.2 | eBioScience |
| CD11b | PerCP-Cy5.5 | M1/70 | Biolegend |
| CD11b | PE-Cy7 | M1/70 | Biolegend |
| XCR1 | PE | ZET | Biolegend |
| CD40 | PE-Dazzle595 | 3/23 | Biolegend |
| CD40 | Pacific blue | 3/23 | Biolegend |
| TCR-β | PE-Cy7 | H57-597 | Biolegend |
| CD4 | FITC | RM4-5 | Biolegend |
| CD8 | APC | 53-6.7 | BD Biosciences |
| CD8 | PerCP-Cy5.5 | 53-6.7 | Biolegend |
| CD44 | BV711 | IM7 | Biolegend |
| CD62L | PE | MEL-14 | Biolegend |
| CD38 | BV711 | 90 | BD OptiBuild |
| CD38 | Pacific Blue | 90 | Biolegend |
| CD101 | PE | Moushi101 | Invitrogen |
| CD101 | Alexa Fluor647 | 307707 | BD Biosciences |
| CD25 | APC | PC61.5 | eBioScience |
| FoxP3 | Alexa Fluor488 | 150D/E4 | eBioscience |
| FoxP3 | PerCP-Cy5.5 | FJK-16s | eBioscience |
| CD274 | PE | HMIH5 | BD Biosciences |
| CD274 | APC | 10F.9G2 | Biolegend |
| IFNAR | PE | MAR1-5A3 | eBioscience |
